# Translation Initiation Factor AteIF(iso)4E Is Involved in Selective mRNA Translation in Arabidopsis Thaliana Seedlings

**DOI:** 10.1371/journal.pone.0031606

**Published:** 2012-02-20

**Authors:** Ana Valeria Martínez-Silva, César Aguirre-Martínez, Carlos E. Flores-Tinoco, Naholi D. Alejandri-Ramírez, Tzvetanka D. Dinkova

**Affiliations:** Departamento de Bioquímica, Facultad de Química, Universidad Nacional Autónoma de México, Distrito Federal, México; University of Hyderabad, India

## Abstract

One of the most regulated steps of translation initiation is the recruitment of mRNA by the translation machinery. In eukaryotes, this step is mediated by the 5′end cap-binding factor eIF4E bound to the bridge protein eIF4G and forming the eIF4F complex. In plants, different isoforms of eIF4E and eIF4G form the antigenically distinct eIF4F and eIF(iso)4F complexes proposed to mediate selective translation. Using a microarray analysis of polyribosome- and non-polyribosome-purified mRNAs from 15 day-old *Arabidopsis thaliana* wild type [WT] and eIF(iso)4E knockout mutant [*(iso)4E-1*] seedlings we found 79 transcripts shifted from polyribosomes toward non-polyribosomes, and 47 mRNAs with the opposite behavior in the knockout mutant. The translationally decreased mRNAs were overrepresented in root-preferentially expressed genes and proteins from the endomembrane system, including several transporters such as the phosphate transporter *PHOSPHATE1* (*PHO1*), Sucrose transporter 3 (*SUC3*), ABC transporter-like with ATPase activity (*MRP11*) and five electron transporters, as well as signal transduction-, protein modification- and transcription-related proteins. Under normal growth conditions, eIF(iso)4E expression under the constitutive promoter 35 S enhanced the polyribosomal recruitment of *PHO1* supporting its translational preference for eIF(iso)4E. Furthermore, under phosphate deficiency, the PHO1 protein increased in the eIF(iso)4E overexpressing plants and decreased in the knockout mutant as compared to wild type. In addition, the knockout mutant had larger root, whereas the 35 S directed expression of eIF(iso)4E caused shorter root under normal growth conditions, but not under phosphate deficiency. These results indicate that selective translation mediated by eIF(iso)4E is relevant for Arabidopsis root development under normal growth conditions.

## Introduction

Translation initiation factor eIF4E binds to the cap structure (7mGpppN, where N is any nucleotide) present at the 5′end of most eukaryotic mRNAs. Through its high affinity binding to the bridge protein eIF4G, this factor participates in the mRNA recruitment for translation. eIF4G interacts with the multi-subunit complex eIF3 bringing together the mRNA and the 43 S initiation complex formed by eIF3, the ternary complex (eIF2-Met-tRNAMet-GTP), the 40 S ribosomal subunit and other initiation factors [1]. It also recruits the RNA helicase eIF4A to unwind secondary structures in the 5′ untranslated region (5′UTR) of the mRNA, and the poly(A) binding protein (PABP) allowing the mRNA circularization for efficient translation re-initiation. In eukaryotes, more than 95% of protein synthesis is initiated involving the cap structure of mRNAs [2] thus making the initiation of step of translation the most controlled event in the process [1].

A change in initiation efficiency has a strong influence on the protein content (quantitative regulation) and the relative levels of different proteins (qualitative regulation). The cap-binding protein, eIF4E, has highly conserved amino acids across all eukaryotic organisms that interact with the 5′ cap structure of mRNAs [3]. eIF4G interacts with eIF4E through a YXXXXLΦ motif (where Φ is any hydrophobic amino acid) and formation of the eIF4G/eIF4E complex (eIF4F) improves the ability to bind the 5′ cap, forming a stable eIF4F-mRNA complex [4], [5], [6]. eIF4E primarily functions in the initiation of translation as part of the eIF4F complex; however, the sequestration of eIF4E may also act to specifically repress translation of mRNAs [7]. During the last few years it became evident that through binding to specific proteins and the cap of mRNAs, eIF4E participates in the nucleo-cytoplasmic transport, translational repression, and turnover of mRNA [7]. The interaction between the cap and the translational machinery may be prevented by the binding of eIF4E to other cellular proteins through the same domain used for its interaction with eIF4G. By such means cells could modulate either the global translation levels, or specific mRNA recruitment [8].

Multiple eIF4E family members have been identified in a wide range of organisms that include plants, flies, mammals, frogs, birds, nematodes, and fish [3], [9]. These members have been classified into three families: eIF4E-I, eIF4E-II and eIF4E-III [3]. This classification was done according to the conservation of Trp 43 and Trp 56 (numbering according to the human eIF4E-1 sequence) in the protein. Members from class I present Trp43 and Trp 56 conserved, while those from Class II have both residues substituted by Tyr or Phe and Class III only have substituted Trp 56 by Tyr, Phe or Cys. Some eIF4E family members have altered cap-binding affinities or interactions with eIF4G and other proteins, providing clues to their physiological roles. It has been suggested that each organism has at least one class I eIF4E that is ubiquitous and constitutively expressed to carry out general translation and that the other family members are involved in specialized functions [9].

Some eIF4E proteins are required only during particular developmental stages or under particular stress conditions. For example, Caenorhabditis elegans expresses four different class I eIF4E proteins; however, only IFE-3 is essential for survival [10]. IFE-1 is required during spermatogenesis [11] and IFE-2 is relevant for longevity and response to oxidative stress [12] and its mutation produces temperature-sensitive defects in meiotic crossover [13]. The single class II family member IFE-4 is involved in proper egg laying [14]. In Schizosaccharomyces pombe, there are two class I eIF4E proteins, eIF4E-1 and eIF4E-2, but eIF4E-2 particularly functions under stress conditions such as nutrient depletion, high temperatures, and high salt, to arrest cell growth and division [15]. In Drosophila melanogaster, there are seven different eIF4E genes encoding eight isoforms (seven belonging to class I and one to class II). All of them are able to bind cap, but a specialized function has been demonstrated for some [16], [17].

In plants, three eIF4E family members have been reported: eIF4E (class I), eIF(iso)4E (plant-specific, class I), and nCBP (novel cap binding protein, class II). The eIF(iso)4E protein interacts with a particular eIF(iso)4G forming the unique plant eIF(iso)4F complex [18]. eIF4F and eIF(iso)4F complexes show selectivity in the recognition of mono and di-methylated cap structures, as well as in in vitro translation of 5′UTR structured mRNAs. In most plant species, eIF(iso)4E shows about 50% amino acid identity with eIF4E and the relative abundance of each protein varies depending on the developmental stage and the plant tissue.

In Arabidopsis, the eIF(iso)4E transcript and protein are more abundant in roots, floral organs and tissues under development [19], [20]. In maize, the eIF(iso)4E protein is present at higher levels than eIF4E in non-germinated seeds [21]. The corresponding transcript is efficiently translated upon imbibition to maintain constant and high levels during the first 24 h of germination, whereas eIF4E levels increase toward germination completion [22]. In addition, each Class I Zea mays eIF4E family member displays selective translational activity on the pool of mRNAs stored in the quiescent embryonic axes [23], [24]. Other plant reports have indicated that the overall ability of the eIF4F and eIF(iso)4F complexes to support translation of individual mRNAs is readily distinguishable [5], [25].

In Arabidopsis thaliana, eIF4E has three genes: eIF4E1 (At4g18940), eIF4E2 (At1g29590) and eIF4E3 (At1g 29550), whereas eIF(iso)4E has one gene (At5g35620). eIF4E1 appears to be the primary form as eIF4E2 and eIF4E3 transcripts show very low expression. Depletion of eIF(iso)4E in Nicotiana tabacum and Arabidopsis thaliana, induces higher eIF4E expression, probably to compensate its function in general translation initiation. Although no obvious developmental phenotype was reported for an eIF(iso)4E knockout (KO) mutant in A. thaliana, the absence of this protein conferred resistance to infection by several potyviruses [26]. The eIF(iso)4F large subunit [eIF(iso)4G], is encoded by two different genes in A. thaliana, eIF(iso)4G1 (At5g57870), and eIF(iso)4G2 (At2g24050). Double mutants on these genes are significantly affected in their growth and reproduction indicating an essential function for the eIF(iso)4F complex during plant development [27]. However, single mutants develop similar to wild type plants under control growth conditions and confer specific viral resistance [28]. These data suggest that in addition to general translation, and their role in the eIF(iso)4F complex, each subunit may be part of specific regulatory mechanism for gene expression during plant development.

In this work we asked whether the absence of eIF(iso)4E has a discriminatory activity in mRNA translation during early Arabidopsis thaliana plant development. The previously characterized KO insertion mutant, AteIF(iso)4E-1(Duprat et al., 2002), hereafter called (iso)4E-1, was used in microarray transcriptional and translational profiling to compare gene expression to wild type (WT; Col-0) 15 day-old plants. Significantly changed genes at the translational level were genes preferentially expressed in roots and the endomembrane system. Analysis on selected genes indicated that the PHO1 transcript is present in polyribosomes of 15 day-old seedlings, but shifted towards free ribonucleoprotein fractions in the (iso)4E-1 mutant. In addition, the (iso)4E-1 mutant primary root was consistently larger at the same developmental stage compared to WT. Under phosphate limitation stress, the PHO1 protein levels were decreased in (iso)4E-1 and increased in an eIF(iso)4E overexpressing transgenic line as compared to WT. These results suggest that the presence of both isoforms, eIF4E and eIF(iso)4E, in a particular proportion is required for selective translation during normal plant growth, as well as in the phosphate limitation stress.

## Results

### Molecular characterization of the mutant lines (iso4E-1) and (iso4E-2)

**Figure 1:**
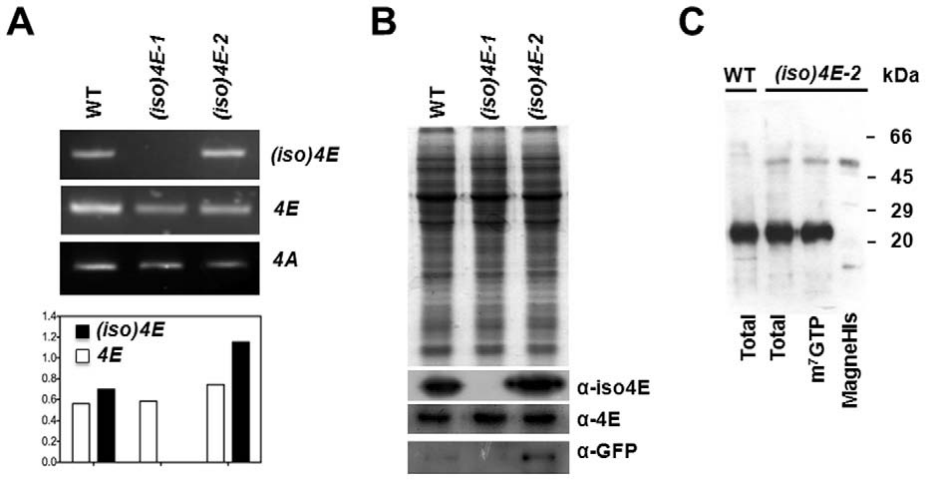
Molecular characterization of *AteIF(iso)4E* mutant plants. Plants from wild type Col-0 [WT], knockout [*(iso)4E-1*] and *35S:eIF(iso)4E:GFP* [*(iso)4E-2*] lines were grown on Gamborg's B5 media for 15 days and total RNA or proteins were extracted from whole seedlings. **A.** Amplification by RT-PCR indicates that *eIF(iso)4E* mRNA is absent in *(iso)4E-1* and increased about 2 fold in *(iso)4E-2*, whereas eIF4E mRNA levels remained similar in all lines. The transcript level of *eIF4A* was used to correct for RNA loading. **B.** Fifty micrograms of total proteins extracted from 15 day-old Arabidopsis seedlings were separated by SDS-PAGE and immunoblotted against eIF(iso)4E, eIF4E and GFP using specific antibodies. The absence of a 22 kDa band corresponding to eIF(iso)4E is observed in the *(iso)4E-1* mutant, whereas a 25 kDa band corresponding to eIF4E is increased. The detection of GFP indicates the presence of a 55 kDa band corresponding to the fusion protein eIF(iso)4E-GFP in the *(iso)4E*-2 transgenic line. **C.** Protein extracts from *(iso)4E-2* 15 day-old seedlings were subjected to m^7^GTP-Sepharose (GE Healthcare) or MagneHis system (Promega) purification. One fifth of the eluted proteins from each was separated by SDS-PAGE together with the total extracts from WT and *(iso)4E-2* plants and immunoblotted against Arabidopsis eIF(iso)4E. A 55 kDa band corresponding to the eIF(iso)4E-GFP fusion carrying a C-terminal His tag was observed in *(iso)4E-2* total extract, and the m^7^GTP- or MagneHis-purified fractions, but not in WT total extract. The 22 kDa band corresponding to native eIF(iso)4E was detected in WT and *(iso)4E-2* total extracts, as well as in the m^7^GTP-bound fraction.

To access the functional relevance of the *Arabidopsis thaliana* eIF(iso)4E protein, a previously reported KO mutant, *(iso)4E-1*, was used [26]. This mutant contains a transposon insertion (*dspm*) bearing the glufosinate ammonium (BASTA) resistance gene into the second exon of *At5g35620* rendering no transcript or protein for eIF(iso)4E. To confirm the stability of the insertion, mutant seedlings were grown in the presence of BASTA and analyzed at RNA and protein levels (Figure 1). No *eIF(iso)4E* RNA or protein was detected for *(iso)4E-1*, whereas the eIF4E protein was increased in agreement with [26].

Additionally, we generated three Arabidopsis transgenic lines, *(iso)4E-2*, *-3*, and *-4*, expressing eIF(iso)4E fused to a GFP tag at the carboxy terminus in the Col-0 WT background (Figure S1, panels A, B). The fusion was separated by 16 amino acids to allow independent folding of the eIF(iso)4E and GFP domains. A structural modeling of the fusion protein indicated no significant alteration in the cap-binding pocket or the surface of eIF(iso)4E expected to interact with eIF4G (Figure S2).The GFP fusion allowed the eIF(iso)4E localization in the transgenic plants (Figure S3). The fusion protein of approximately 55 kDa reacted against Arabidopsis eIF(iso)4E and GFP antiserums and was able to bind m^7^GTP-Sepharose (Figure 1C), indicating its functionality in binding the capped mRNAs. The three independent transgenic lines expressing eIF(iso)4E-GFP showed increased levels of the eIF(iso)4E RNA as compared to WT in 15 day-old seedlings ranging from 2.5 to 3.5 times (Figure S1, panel C). Therefore, these plants express eIF(iso)4E above its normal level. For some of the further experiments, only the *(iso)4E-2* line was selected, whereas for others the three transgenic lines were included. Additionally, a transgenic line expressing under a 35 S promoter eIF4E (*4E-ϕ*) fused to a GFP tag at the carboxy terminus in the Col-0 WT background was used as control for some experiments (Figure S3).

### Changes in mRNA association with non-polyribosomal (NP) and polyribosomal (P) fractions in (iso)4E-1 null mutant line

Previous reports describing *(iso)4E-1* did not report any obvious phenotype in leaf development and morphology, plant size, flowering and fertility, inferring that the eIF4E protein is able to function in place of eIF(iso)4E. However, even though the two proteins belong to Class I and might perform partially redundant function, changes at the level of selective translation of mRNAs due to the lack of either cap-binding protein may not be ruled out [5], [25], [29]. The polyribosomal distribution of mRNAs in 15 day-old WT Col-0 and *(iso)4E-1* mutant seedlings was analyzed using Arabidopsis Oligonucleotide Microarray slides obtained from the University of Arizona (29,000 oligonucleotides AROS V.3.0). The developmental stage was chosen on the basis of enriched eIF(iso)4E expression in actively growing tissues [19], [30]. On the other hand, at the time of the microarray experiment design, proteomic data from *Arabidopsis thaliana* tissues were not available. The sedimentation profiles on 20–60% sucrose gradients [31] monitored at A_260_ indicated no global differences between wild type and *(iso)4E-1* mutant plants (Figure S4). The fractions from each profile were pooled as non-polyribosomes (NP), containing free ribonucleoprotein complexes, ribosomal subunits 40 S/60 S, monosomes 80 S (fractions 1–5) or polyribosomes (P) containing two or more ribosomes (fractions 6–12). During translation, mRNAs that are initiated efficiently are found on polysomes, whereas a decrease in initiation rate relative to elongation results in a shift towards non-polysome fractions [32].

Using the z-score transformation of two normalized microarray data for independent biological samples (http://www.ncbi.nlm.nih.gov/geo/query/acc.cgi?acc=GSE29386), significantly changed genes in the mutant NP and P fractions were found on a cutoff of 1.5 fold either up or down. According to this criterion 1474 transcripts decreased and 1621 increased in NP, whereas 1558 transcripts decreased and 1491 increased in P fractions of *(iso)4E-1* plants. However, from these only 79 transcripts decreased their presence in P with a concomitant increase in NP (Group I, translationally inhibited, Table S2) and 47 transcripts increased in P with a concomitant decrease in NP (Group II, translationally enhanced, Table S3) in the absence of eIF(iso)4E.

**Figure 2:**
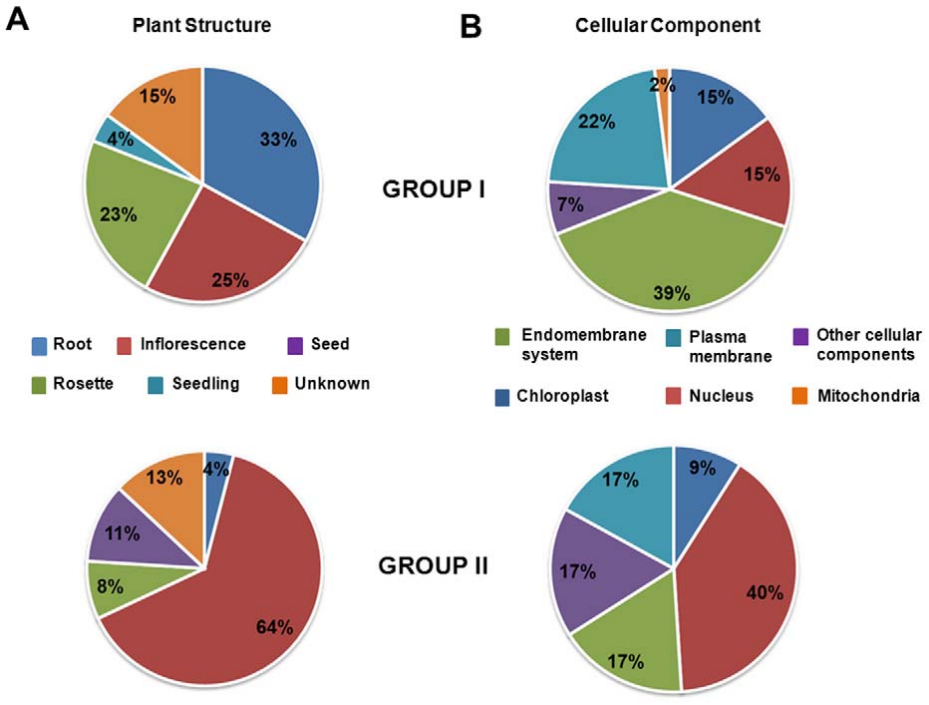
Transcripts differentially translated between WT and *(iso)4E-1* plants are enriched in genes with particular expression and sub-cellular localization patterns. Classification was made according to significant changes (−1.5>z-score>1.5) in both, NP and P, fractions obtained from sucrose gradients (Figure S4). Group I is represented by mRNAs with decreased in P and increased in NP levels; Group II includes mRNAs increased in P and decreased in NP. A. Each gene, within the correspondent group, was classified according to the plant structure reported with the highest transcript level using the AtGenexpress Visualization tool (Schmid *et* al., 2005; TAIR, http://www.arabidopsis.org/). B. Classification of translationally changed mRNAs in *(iso)4E-1* according to their subcellular distribution (TAIR, http://www.arabidopsis.org/) . For the calculation of percentages shown in the graph, only proteins with known subcellular distribution were considered.

The 79 translationally inhibited transcripts represent potential candidates to be preferentially recruited by eIF(iso)4E for their translation. Of these mRNAs, ≈33% were preferentially expressed in roots and ≈25% in inflorescences (Figure 2A), similar to the expression pattern reported for eIF(iso)4E mRNA (Figure S5). On the other hand, the 47 transcripts translationally enhanced in the absence of eIF(iso)4E, which are potential candidates to be preferentially recruited by eIF4E for their translation, were strikingly enriched in mRNAs expressed in inflorescence (64%). According to the gene product cellular component, Group I (translationally inhibited) was also enriched in endomembrane system proteins, whereas Group II (translationally enhanced) included a high number of nuclear factor genes. In Group I of translationally decreased mRNAs, several transporter proteins were found including the phosphate transporter *PHOSPHATE 1* (*PHO1*), the ABC transporter-like *Arabidopsis thaliana* multidrug resistance associated protein 11 (*MRP11*) with ATPase activity, the sucrose transporter 3 (*SUC3*) and five electron transporters (*At1g12570*, *At5g44410*, *At2g20270*, *At3g20950* and, *At4g08455*). Additionally, other plasma membrane and endomembrane system proteins involved in signal transduction and post-translational modification of amino acids were abundant in this group. Genes belonging to Group II included many transcription factors and DNA-binding proteins, as well as two histone acetyl transferases (*At5g56740* and *At1g77540*), translation initiation factor 3 k (*eIF3k*) and the ribosomal protein L12 (*rpL12*). Hence, several of the constituents of Group II may be associated with important changes at transcription level in the *(iso)4E-1* KO mutant.

To separate the effect on transcription from that on translation, Groups I and II of translationally affected mRNAs included only those remaining unchanged at steady-state (total) RNA level. However, the transcriptome analysis accessed by total RNA analysis of the same plants by microarrays, indicated that 496 genes were up-regulated in the *(iso)4E-1* mutant, whereas 296 were repressed with a cutoff of z-score greater than 2 either up or down. Most of the mRNAs that changed corresponded to metabolism and gene information related proteins (Figure S6). Such findings are in accordance with the changes observed in the polyribosomal distribution of transcription factors or DNA binding proteins in the absence of eIF(iso)4E.

Several randomly selected gene IDs from Groups I and II were chosen to confirm the microarray data by real time reverse transcription PCR (qRT-PCR) on a different biological sample. As observed in Table 1, in a qRT-PCR analysis 20 out of 23 assayed mRNAs reproduced the changes observed in the microarray analysis. Three mRNAs from Group I showed a distribution pattern different to the one obtained from microarray data. This suggests about an 87% confidence level for the microarray analysis. For further analysis, only the mRNAs that were validated by qRT-PCR were considered.

**Table 1:** Quantitation of specific mRNA changes in *(iso)4E-1* Arabidopsis plants.

Accession	Function/Gene Name	Relative mRNA levels					Change
		*T* a	*NP* a	*P* a	*NP* b	*P* b	
*At3g23430*	Phosphate transport (*PHO1*)	0.59	1.58	−2.3	1.9	−15.8	*Decrease*
*At4g02950*	Ubiquitin family protein	−0.51	2.80	−2.2	−3.1	−6.8	*Decrease*
*At4g20340*	Transcription initiation factor (*TFIIE*)	1.15	2.95	−2.8	1.0	−6.2	*Decrease*
*At3g55580*	Regulator of chromosome condensation, (*RCC1*)	0.65	2.5	−2.6	−2.6	−4.2	*Decrease*
*At2g02860*	Sucrose transporter 3 (*SUC3*)	0.97	1.6	−1.9	2.2	−3.4	*Decrease*
*At2g30260*	Component of the U2 snRNP complex (*U2B″*)	0.28	2.5	−2.6	5.6	−2.9	*Decrease*
*At1g21630*	Calcium ion binding, EF hand protein	−0.64	3.5	−2.6	3.2	−1.5	*Decrease*
*At2g17630*	Pyridoxal phosphate (PLP)-dependent transferase	1.25	2.5	−2.2	2.8	−1.4	*Decrease*
*At1g78240*	Tumorous shoot development 2 (*TSD2*)	0.88	1.6	−1.6	1.5	−1.0	*Decrease*
*At2g27940*	RING/U-box protein	−0.04	−1.98	1.8	−2.3	15.6	*Increase*
*At2g28600*	P-loop containing nucleoside tri-phosphate hydrolase	0.29	−2.09	1.8.	−1.3	8.1	*Increase*
*At1g68670*	MYB-like transcription factor family	0.55	−1.8	1.8	1.7	4.3	*Increase*
*At4g06746*	Transcription factor ERF/AP2 DREB subfamily A-5 (*RAP2.9*)	1.63	−1.8	2.1	−1.9	1.9	*Increase*
*At3g57600*	Transcription factor ERF/AP2 DREB subfamily A-2 (*ERF/AP2*)	*n.d.*	−2.5	1.7	−1.5	1.9	*Increase*
*At2g05710*	Aconitase (*ACO3*)	−1.95	−3.2	2.6	−13.3	1.8	*Increase*
*At5g61430*	NAC domain containing protein (*NAC5*)	1.92	−1.8	2.3	−4.9	1.7	*Increase*
*At1g6880*	Transcription factor (*BRC2*)	−0.7	−1.7	1.6	−11.6	1.5	*Increase*
*At4g18720*	Transcription factor IIS protein	0.96	−2.7	3.2	−4.3	1.4	*Increase*
*At5g15850*	Transcription factor, constans-like 1 (*COL1*)	*n.d.*	−2.0	1.7	−2.2	1.3	*Increase*
*At4g33250*	Eukaryotic translation initiation factor 3 K (*EIF3K*)	*n.d.*	−1.8	2.6	5.1	4.9	*Increase*
*At5g15630*	Cobra-like4 (*COBL4*)	1.57	2.8	−1.6	6.2	1.4	*l.c.*
*At5g01840*	Ovate family protein 1 (*OFP1*)	0.59	3.6	−2.8	2.3	5.1	*l.c.*
*At1g64580*	Pentatricopeptide repeat-containing protein (*PPR*)	0.86	1.6	−2.0	2.7	6.3	*l.c.*

a mRNAs significantly (p<0.001) changed (±1.5 cutoff) at both polyribosomal (P) and non-polyribosomal (NP), but not at total (T) RNA levels were considered for quantitation by real time RT-PCR (qRT-PCR). Selection was made randomly.

b Real time RT-PCR was performed with a different biological sample at the same time point as microarrays (15 day-old seedlings). The results were normalized according to eIF4A control gene and show fold of change of the mRNA level in NP and P separately.

*n.d.*: not detected.

*l.c.*: lack of correspondence between microarray and qRT-PCR measurements.

### Untranslated region features of group I and group II mRNAs

Previous work with plant eIF4E and eIF(iso)4E as part of their corresponding cap-binding complexes has indicated that they might display selection on mRNA recruitment depending on the 5′UTR length and presence of secondary structure [25], [29]. Therefore, we performed computational analysis on mRNAs from Groups I and II to compare their 5′UTR characteristics.

The 5′ and 3′ UTR regions were downloaded from The Arabidopsis Information Resource (TAIR10: 11/17/10 release) and the longest expressed sequence tag was selected for each UTR. To avoid partial 5′end sequences, further analysis subtracted the gene accessions listed in the Salk-Stanford-PGEC Arabidopsis ORF gene list [33] and they were used to determine their UTR length and characteristics (Table 2). Noticeably, the 5′UTR length and the presence of stable secondary structures were greater for genes belonging to Group II (with expected preferential translation by eIF4E). This is in agreement with *in vitro* experiments performed years ago with wheat eIF4F and eIF(iso)4F complexes [29]. Furthermore, internal initiation (IRES)-like elements, uORFs and uAUGs were more frequent in the same Group of mRNAs, probably due to the length of the 5′UTR. On the contrary, the 3′UTR length showed similar proportion in Group I and II genes. This analysis confirmed that discrimination between mRNAs by plant eIF(iso)4E and eIF4E probably relies on the 5′UTR length and structure, although particular sequences in the 3′UTR having a role in selectivity should not be ruled out.

**Table 2:** Untranslated region (UTR) analysis for mRNAs changed in their polysomal distribution (Group I and II from Figure 2) in the *(iso)4E-1* mutant.

		Group I^a^	Group II^a^	Random selection^b^
5′UTR	≥100 bp length	33	47	43
	<100 bp length	67	53	57
	secondary structure (ΔG<−20 kcal/mol)	25	47	36
	uAUGs	11.5	14.3	–
	uORFs	10	14.3	–
	IRES-like	7.7	14.3	–
3′UTR	≥100 bp length	89	87	83
	<100 bp length	11	13	17
	CPE	2	0	–

a All data represent percentages of the total of sequences within the group.

b Randomly selected *Arabidopsis thaliana* accessions (80) were evaluated for 5′ and 3′UTR length and secondary structures to support the significance of percentages for Groups I and II.

uAUGs: upstream AUGs; uORFs: upstream open reading frames; IRES: internal ribosome entry site; CPE: cytoplasmic polyadenylation element.

### Effect of expressing AteIF(iso)4E under a 35 S constitutive promoter on the polyribosomal distribution of selected mRNAs

**Figure 3:**
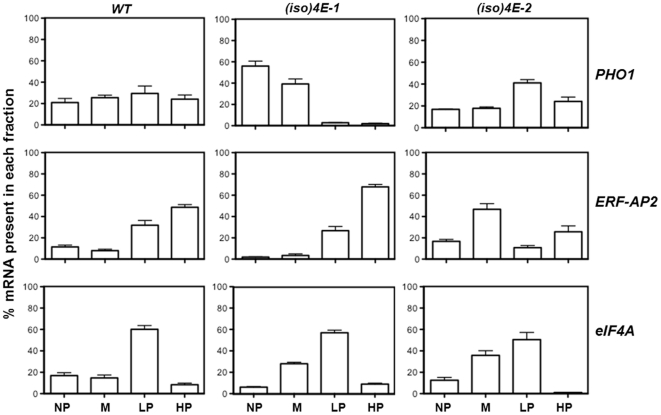
Specific mRNAs show preferential recruitment to polyribosomes depending on eIF(iso)4E levels. The polyribosomal distribution of selected mRNAs was analyzed in the WT, knockout [*(iso)4E-1*] and overexpressing [*(iso)4E-2*] lines at the 15 day-old seedling stage by qRT-PCR. The percentage of mRNA found in free RNP (NP), monosomes (M), low polyribosomes (LP) and high polyribosomes (HP) fractions (collected according to Figure S4) was calculated as described in Material and Methods. *PHO1* reduced its presence in LP and HP fractions in the absence of eIF(iso)4E and increased in LP above the WT level in seedlings overexpressing this eIF4E family member. Transcription factors *ERF/AP2* shifted toward the HP fraction in *(iso)4E-1* and was slightly enriched in the M fraction in *(iso)4E-2* plants as compared to WT. Translation factor *eIF4A* polyribosomal distribution profiles were similar in all Arabidopsis lines used in this study, indicating that this transcript was not translationally affected by changes of eIF(iso)4E levels. The data represent average of three independent replicates and bars indicate the standard error.

To test whether in the *(iso)4E-2* transgenic line there was an alteration on the polyribosomal distribution of some of the mRNAs shown in Table 1, qRT-PCR was performed on RNA isolated from sucrose gradients performed on different biological samples of 15 day-old seedlings (Figure 3). The transcript corresponding to the translation initiation factor *eIF4A* was used as control mRNA apparently not affected by eIF(iso)4E levels. To evaluate more accurately the distribution of selected mRNAs along the polyribosomal profiles, the NP fraction used for microarray analysis was further divided into monosomes (M) and free ribonucleoproteins, RNPs (NP), whereas the P fraction was divided into low polyribosomes (LP) and high polyribosomes (HP). The mRNA corresponding to the phosphate transporter *PHO1* was shifted to NP and M in the *(iso)4E-1* null mutant whereas it increased in LP and was similar in the HP with respect to WT in the *(iso)4E-2* overexpressing line. This places the *PHO1* mRNA as a strong candidate for eIF(iso)4E dependent translation.

In the absence of eIF(iso)4E, the mRNAs from Group II were modestly enhanced on polysomes (Table 1). Such behavior was reproduced for the transcription factor *ERF/AP2*, which appeared shifted particularly toward the HP fraction in *(iso)4E-1* (Figure 3). On the contrary, in the *(iso)4E-2* overexpressing line this mRNA was increased in the M fraction as compared to the WT profile. These results suggest that the proportion of eIF(iso)4E to eIF4E proteins is relevant for *in vivo* selective mRNA translation.

In the *(iso)4E-1* KO mutant, increased eIF4E protein levels have been reported [26]. Therefore, alterations in eIF4E might also account for the observed shifting of *PHO1* and/or *ERF/AP2* factor in the polyribosomal profile of this mutant. We used a transgenic line overexpressing eIF4E to evaluate the polyribosomal distribution of these mRNAs (Figure S7). The *4E-ϕ* line presented a slight *PHO1* shift toward the M fraction as compared to WT and did not show changes for the *ERF/AP2* transcription factor distribution along the polyribosomal profile. This further supports the idea that the absence of eIF(iso)4E in the *(iso4E)-1* null mutant is responsible for the observed downregulation of *PHO1* mRNA translation.

### PHO1 expression at protein level is altered in eIF(iso)4E mutants

**Figure 4:**
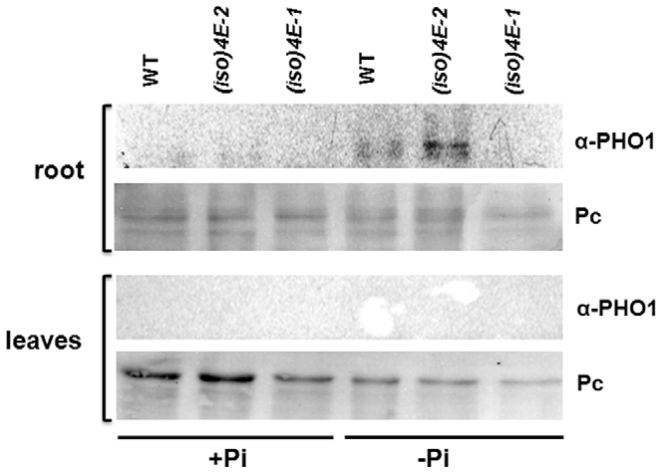
Western blot analysis of PHO1 protein levels in seedlings grown under phosphate sufficiency (+Pi) or deficiency (−Pi). One week -old seedlings from WT, *(iso)4E-1* and *(iso)4E-2* lines were transferred to liquid medium containing 1 mM (+Pi) or 10 µM (−Pi) phosphate and grown for an additional week. After that roots and leaves were carefully separated and total proteins extracted from each tissue. Thirty micrograms of total proteins were used for each sample. A reacting band corresponding to the molecular weight of PHO1 was detected only for roots of WT and the *(iso)4E-2* overexpressing line in the –Pi condition. As protein loading control, a region of the Ponceau stained membrane is shown in the lower panel of each tissue (Pc).

Since PHO1 is a major phosphate transporter required under phosphate (Pi) deficiency in *Arabidopsis thaliana* [34], we hypothesized that eIF(iso)4E might have a role under this particular stress. Therefore, we analyzed *PHO1* expression in 15 day-old WT and mutant seedlings transferred for 10 additional days to no (−Pi) or normal (+Pi) phosphate concentration in the medium. As expected, the *PHO1* transcript level was increased in WT phosphate starved plants (Figure S8), indicating that plants were sensing the imposed stress. Interestingly in both, *(iso)4E-1* and *(iso)4E-2*, mutant lines the *PHO1* mRNA levels were similar to WT in the presence of phosphate, but showed only a modest increase under Pi starvation conditions compared to WT suggesting that there might be an indirect effect of eIF(iso)4E levels on transcription or stability of *PHO1* mRNA in response to phosphate starvation. We further analyzed the PHO1 protein levels by western blot in WT and eIF(iso)4E mutant plants under both, +Pi and –Pi conditions (Figure 4). For these experiments seedlings were separated in roots and leaves, since PHO1 is mainly localized in root tissues [35]. Using 30 µg of total root proteins we were able to detect the PHO1 protein under –Pi condition in WT and the *(iso)4E-2* overexpressing mutant, but not in the *(iso)4E-1* KO mutant. Using the same amount of protein from leaves, PHO1 was not detected in accordance with a previous report using the same antibody [35]. Roots overexpressing eIF(iso)4E showed higher accumulation of PHO1 than WT under phosphate deficiency (Figure 4). However, in *(iso)4E-1* roots the PHO1 protein was undetectable. Under normal growth conditions (+Pi) the PHO1 protein levels remained beyond the western blot sensitivity, even for the *(iso)4E-2* overexpressing mutant. Taking into account these results and the previous data on *PHO1* polysomal distribution by qRT-PCR (Figures 3 and S7) we concluded that eIF(iso)4E is required for selective *PHO1* mRNA translation.

### The primary root elongation is altered in AteIF(iso)4E mutant plants

**Figure 5:**
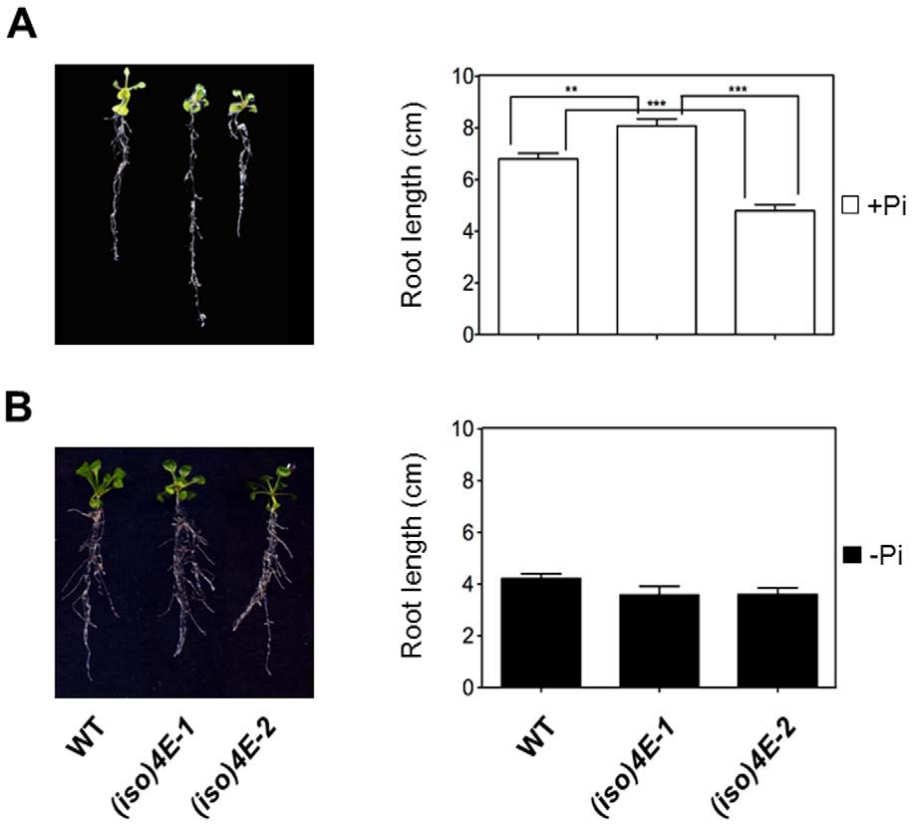
The eIF(iso)4E absence or overexpression is associated with root length alteration in *Arabidopsis thaliana*. Plants from wild type Col-0 [WT], knockout [*(iso)4E-1*] and overexpressing [*(iso)4E-2*] lines were grown on Gamborg's B5 for 15 days and then transferred for 10 days to Hoagland medium supplemented with (+Pi) or without (−Pi) phosphate in squared Petri dishes placed vertically in a growth chamber. Data are shown as the mean of 9 biological replicates with 3 technical replicates of each one, with error bars indicating the standard error. One way ANOVA with Tukey's Multiple Comparison Test with a p value<0.05 was used. Significant differences in primary root length between the 3 lines are indicated by asterisks (**, p<0.01; ***, p<0.001). **A.** In the presence of phosphate, the *(iso)4E-1* primary root was significantly larger than WT (p<0.01), whereas *(iso)4E-2* was significantly shorter than WT and *(iso)4E-1* (p<0.001). **B.** After 10 days under phosphate deficiency, the primary root of all three lines was significantly shorter than the correspondent to plants grown in control conditions and did not display significant differences between lines.

Since the PHO1 protein is involved in phosphate uptake and is predominantly expressed in *Arabidopsis* seedling root and lower hypocotyl, its altered expression levels in *AteIF(iso)4E* mutants may affect the root architecture under normal growth conditions. Analysis of WT and mutant plants, indicated that 25 day-old *(iso)4E-1* seedlings [grown for 15 days on Gamborg's B5 and transferred for 10 additional days on Hoagland (+Pi)] had increased primary root length by 1 cm when compared to WT (Figure 5A). On the contrary, *(iso)4E-2* had a shorter length by approximately 2 cm at the same time. The observed differences were statistically significant at p<0.001. The shorter root phenotype was reproducibly observed for three independent transgenic lines expressing AteIF(iso)4E under a constitutive S35 promoter, ruling out that the altered root growth might be a result of a particular transgene insertion site (Figure S9).

Inhibition of primary root growth is a characteristic plant phosphate starvation response [36]. Therefore, we tested the behavior of eIF(iso)4E mutant seedlings under phosphate deficiency compared to WT. Strikingly, there were no obvious differences in primary root length between WT and either *(iso)4E-1* or *(iso)4E-2* seedlings in phosphate starvation (Figure 5B). Statistical analysis indicated that the low phosphate concentration reduced more the primary root growth in *(iso)4E-1* than WT (p<0.001), whereas there was only a slight reduction in growth of an already shorter *(iso)4E-2* root (p<0.05). Hence, the effect on root growth observed under altered eIF(iso)4E levels was surpassed by the condition of Pi deficiency.

## Discussion

The presence of multiple eIF4E family members is widespread in eukaryotes including plants. Two different proteins, eIF4E and eIF(iso)4E, have been shown to participate in plant translation initiation through cap-binding of the mRNAs and recruiting other initiation factors [18]. Whether their role is redundant or specialized has remained obscure. Here, the physiological relevance of eIF(iso)4E was analyzed using two Arabidopsis mutants, one containing an insertion in the second exon of the gene with null expression of the protein, the other expressing a fusion protein eIF(iso)4E-GFP under a constitutive promoter in a WT background. The KO mutant, (iso)4E-1, was previously characterized [26] indicating no obvious phenotype except for enhanced potyviral resistance. Additionally, eIF4E was reported at increased levels in this mutant suggesting that it may fulfill the lacking eIF(iso)4E function. In this work we identified differentially translated mRNAs in the (iso)4E-1 mutant by examining non-polyribosomal (NP), polyribosomal (P) and total RNA fractions using DNA microarray and qRT-PCR. Specifically, 79 transcripts decreased their levels in P fractions with a concomitant increase in the NP fractions (Group I) and 47 transcripts increased in P simultaneously with a decrease in NP (Group II) without changes in their total RNA levels. Only one mRNA in group II showed a change in its steady-state RNA level in addition to a change in polyribosomal distribution. It was excluded from the analysis since in this case we could not separate the effect of transcription from that of translation. However this mRNA and others, whose translational behavior might have been obscured by important transcriptional changes in the mutant were not included in this study and could also be candidates for eIF(iso)4E selective translation.

The quantitative RT-PCR analysis performed for a limited number of genes confirmed a wide range of the changes between NP and P fractions (Table 1). These results indicate that the presence of eIF(iso)4E, in addition to eIF4E, is required in the plant to achieve translational recruitment of some mRNAs. A computational analysis of the UTR regions of transcripts from Group I and II confirmed that they differ principally in their 5′UTR length and secondary structure. This is in accordance with previous in vitro studies showing that eIF(iso)4E and eIF4E, each in their correspondent eIF(iso)4F and eIF4F complex, are able to discriminate the length and frequency of secondary structure in the 5′UTR of a mRNA [29] and consequently to promote translation initiation at different rates [5], [25]. The fact that in the absence of eIF(iso)4E some mRNA recruitment to polyribosomal fractions become enhanced indicates that they mostly depend on eIF4E and the correspondent eIF4F Cap-binding complex for translation initiation. Translation initiation factors, such as eIF4A, eIF4B, eIF3, and others are shared between eIF4F and eIF(iso)4F for translation initiation. Since their presence per se in the initiation complex may also have different impact on selective mRNA translation, an enrichment of these factors particularly for the eIF4F complex in the absence of eIF(iso)4E might also account for the observed mRNA translation enhancement.

According to the developmental stage at which the microarray analysis was performed, it was interesting to notice that in (iso)4E-1 most of the translationally affected transcripts represented root expression and endomembrane system proteins, whereas those translationally enhanced were inflorescence-expressed and transcription factors. Available data on eIF4E and eIF(iso)4E expression patterns are indicative of preferential, albeit not exclusive, localization of the eIF(iso)4E protein in young roots, whereas eIF4E is more ubiquitously distributed [37]. This may explain why the absence of eIF(iso)4E mostly affected translation of mRNAs expressed in roots. The candidate for eIF(iso)4E preferential translation, PHO1, showed enhanced translation in the (iso)4E-2 mutant, where eIF(iso)4E was expressed under a constitutive S35 promoter, whereas ERF/AP2 whose translation was increased in the absence of eIF(iso)4E, slightly decreased its presence in polyribosomal fractions. This observation supports the notion that some mRNAs may be particularly sensitive to eIF(iso)4E levels when they have to compete with other transcripts for their translation. In addition, a particular localization of each eIF4E class I protein at the tissue and/or sub-cellular level may account for the observed translational preference of mRNAs. The last has been the case of eIF4E family members from other organisms, such as Caenorhabditis elegans [11], [14] and Drosophila melanogaster [16].

Since several root-expressed mRNAs changed their translation profile in the absence of eIF(iso)4E, a logical assumption was to look more closely to the root morphology of 15 day-old Arabidopsis WT and mutant seedlings. The previous (iso)4E-1 mutant characterization had not observed the phenotype of roots [26] and we didn't expect obvious defects in root development, since it is closely related to leaf development which proceeds normally in the absence of eIF(iso)4E. However, a consistent and statistically significant larger primary root was observed for 15 day-old seedlings and more lateral root growth was often detected. In addition, for three independent lines expressing eIF(iso)4E under a constitutive promoter, a statistically significant shorter primary root was characteristic. Searching for genes involved in root elongation within the genes corresponding to mRNAs whose polyribosomal distribution was changed in the microarray analysis of (iso)4E-1, we found several accessions in Group I related to altered primary root elongation. These include PHO1, the BTB/POZ domain containing protein At4g08455, the EXTENSIN 3 (EXT3) and the polysaccharide binding protein COBRA-LIKE 4 (COBL4), although the changes of COBL4 were not reproduced in the qRT-PCR experiment. In addition, other proteins whose translation was enhanced in the mutant (Group II) may have contributed to the observed phenotype under normal growth conditions.

The change in specific mRNA polyribosomal distribution in (iso)4E-1, such as that corresponding to PHO1, lead us to propose that eIF(iso)4E might be particularly required during stress conditions such as phosphate deficiency, where PHO1 protein levels are crucial [34]. In WT plants, the PHO1 protein is particularly detected in roots when phosphate availability becomes limiting ([35]; Figure 4). We found that the (iso)4E-1 roots did not show the characteristic PHO1 protein increase upon the –Pi stress, whereas (iso)4E-2 presented higher than WT PHO1 protein levels. This further supports the role of eIF(iso)4E in selective PHO1 mRNA translation.

The availability of soil nutrients can alter root development [36]. Phosphate deficiency induces the formation of lateral roots and inhibits root elongation to achieve a dense, highly branched root system. Inhibition of the cell cycle, low auxin concentrations in the root apical meristems and the expression of phosphate transporter genes result in a root system highly adapted for efficient uptake of Pi. Contrary to our observation on root development in the presence of Pi, we found that the (iso)4E-1and (iso)4E-2 primary root length was similar to WT after 10 days in the absence of Pi. In addition, none of the eIF(iso)4E mutants showed altered root hair formation with respect to WT roots under control or phosphate limitation conditions. These observations indicate that the regulatory mechanisms in response to phosphate limitation overrule the alterations imposed by a possible unusual eIF4E/eIF(iso)4E proportion in the root.

Regarding a correlation between the *PHO1* altered translational distribution in *(iso)4E-1* and *(iso)4E-2* mutants and the observed root phenotype, it may exist or not. A couple of recent publications have shown very interesting new concepts about the *PHO1* role in the Pi deficiency related-phenotype and an influence of its transcript expression levels on Pi efflux and cell growth [35], [38]. Two major observations derived from these reports: (1) the plant response to Pi deficiency involves a major gene expression reprogramming responsible for the observed phenotypes under this stress which is independent of the *PHO1* levels, and (2) an increase in the *PHO1* transcript levels in shoot induces major Pi efflux from the cell toward the vascular system with a concomitant cell growth reduction. Taking this into account, changes in *PHO1* translational levels under normal growth conditions in the eIF(iso)4E mutants used in this study may have contributed, in addition to other gene expression alterations, to the root growth phenotype.

Overall the results obtained here indicate that selective translation by altering the eIF(iso)4E levels and correspondingly its proportion relative to eIF4E is much more complex, than simply assuming that each factor specifically recruits different group of mRNAs to the ribosome. In addition to the preference of each factor for a particular 5′UTR, eIF4G or eIF(iso)4G within the eIF4F and eIF(iso)4F cap-binding complexes, other translation factors or regulatory proteins, probably account for the plasticity in the translation profile of a given mRNA in response to stress conditions.

## Methods

### Plant materials

Seeds of wild type (WT) *Arabidopsis thaliana* ecotype Columbia (Col-0) were obtained from the Arabidopsis Biological Resources Center (ABRC) at Ohio State University. The transposon insertion mutant for the eIF(iso)4E gene, *(iso)4E-1*, was kindly donated by Dr.Christophe Robaglia from CNRS-CEA Université de la Mediterranée, France. Transgenic lines expressing eIF(iso)4E as GFP fusion protein under the constitutive Cauliflower Mosaic Virus (CaMV) 35 S were generated using the Gateway® cloning technology (Invitrogen Corp., Carlsbad, CA, USA). The DNA coding region of *eIF(iso)4E* or *eIF4E* was first amplified by PCR with the appropriate primers and then cloned into pTOPO-D vector (Invitrogen). Finally, the coding region was introduced by recombination with LR clonase (Invitrogen) into the pEarleyGate103 binary vector [39] as a destination vector. Positive recombinant was confirmed by DNA sequencing. The plasmid, bearing the fusion gene and the ammonium glufosinate (BASTA) resistance gene (Figure S1), was introduced into the GV3101 *Agrobacterium tumefaciens* strain, and used to transform Col-0 plants by floral dip transformation [40]. Several independent homozygous lines were isolated by their BASTA resistance and self-fertilized for at least three generations prior to use. The plant line *(iso)4E-2* was selected as representative for the experiments based on mRNA expression levels (Figure S1). The same procedure was followed for eIF4E overexpressing transgenic lines and one of them (*4E-ϕ*) was selected for some experiments as described in results.

### Growth conditions

All reagents were from Sigma-Aldrich Quimica, Mexico unless otherwise stated. Arabidopsis thaliana seeds were surface-sterilized in Tween 20 (0.1%) and sodium hypochlorite 20% for 10 minutes and rinsed 5 times with sterile deionized water. The seeds were spread on sterile 0.7% (w/v) agar (Becton Dickinson, Mexico) containing Gamborg's B5 minimal medium with 1% sucrose. The seeds were stratified at 4°C in the dark (Petri dish wrapped in two layers of aluminum foil) for two days and then grown for 15 days at 20°C under 100 µmol m-2 sec-1 light intensity in a 8/16 hr light/dark photoperiod.

Plants used for protein analysis were grown for one week in a medium containing half-strength Murashige-Schoog (MS), 1% sucrose and 0.6% gellan. Then, seedlings were transferred to a liquid medium containing 1% sucrose, 2.5 mM KNO_3_, 100 µM Ca(NO3)_2_, 1 mM MgSO_4_, 25 µM Fe-EDTA, 14 µM MnCl_2_, 0.5 µM CuSO_4_, 1 µM ZnSO_4_, 70 lM H_3_BO_3_, 0.2 µM NaMoO_4_ and 10 µM NaCl (final pH 5.7) supplemented with either 1 mM (+Pi) or 10 µM (−Pi) KH_2_PO_4_.

For root phenotype studies, seeds were grown in Gamborg's B5 solid medium for two weeks and afterwards transferred to Hoagland medium containing 6 mM KNO_3_, 4 mM Ca(NO_3_)_2_·H_2_O, 2 mM MgSO_4_·7H_2_O, 0.009 mM MnCl_2_·4H_2_O, 0.046 mM H_3_BO_3_, 0.0008 mM ZnSO_4_·7H_2_O, 0.0003 mM CuSO_4_·5H_2_O, 0.0001 mM H_2_MoO_4_·H_2_O, 0.005 g/ml Fe-EDTA and 0.5 mM NH_4_H_2_PO_4_ as a phosphate source (+Pi), or Hoagland medium including all of the above except for the 0.5 mM NH_4_H_2_PO_4_ which was substituted by 0.5 mM (NH_4_)_2_SO_4_ (−Pi) for 10 days.

### Root length analysis

To scan root development, Arabidopsis thaliana seeds were grown in square Petri dishes oriented vertically. At 20 days after germination the plants were carefully removed from the medium and the primary root length was measured using digital ruler (ImageJ). Five plants per line (WT, (iso)4E-1 and (iso)4E-2 mutants) were tested per experiment, and the experiment was repeated 9 times. To analyze the effect of phosphate deficiency on root development, Arabidopsis seeds were germinated on Gamborg's B5 for 2 weeks and then transferred to Hoagland medium containing phosphate (+Pi) or without phosphate (−Pi) as described above. Five plants per line were tested per experiment, and the experiment was repeated 5 times. All data were analyzed by one way ANOVA with Tukey's Multiple Comparison Test using a p value<0.05.

### Protein extraction and Western blot analysis

Total protein extracts were prepared by homogenizing frozen leaf or root material in extraction buffer [100 mM HEPES pH 7.5, 5% v/v glycerol, 50 mM KCl, 5 mM EDTA, 5 mM NaF, 0.1% v/v Triton X-100, 1 mM DTT and one Complete EDTA-free protease inhibitor cocktail tablet (Roche Molecular Diagnostics, Pleasanton, CA, USA) per 50 ml] in the ratio of 2 g of tissue to 3 ml buffer. The samples were centrifuged at 13 000 *g* for 15 min and clear supernatants were collected. The protein concentration was determined using Bradford reagent (Bio-Rad Laboratories, Inc. Hercules, CA) and bovine serum albumin (BSA) as the standard. For cap-binding protein purification, the protocol from [20] was followed. The eIF(iso)4E-GFP fusion protein was purified using the MagneHis system (Promega Corp., Madison, WI, USA).

Thirty micrograms of total protein were separated by SDS-PAGE and electroblotted onto polyvinylidene fluoride (PVDF) membrane (Millipore Corp., Billerica, MA, USA), which was blocked with 5% (w/v) milk in PBS-Tween and incubated with the primary antibody overnight at 4°C. Rabbit antibodies to Arabidopsis eIF4E and eIFiso4E were kindly provided by Dr. Karen Browning (Department of Chemistry and Biochemistry, University of Texas at Austin, TX) and used at 1∶10,000 dilution. Rabbit antiserum against Arabidopsis PHO1 was kindly donated by Prof. Yves Poirier (Département de Biologie Moléculaire Végétale, Université de Lausanne, Switzerland) and used at 1∶1,000 dilution. The secondary antiserum was used at 1∶10,000 dilution and incubation for 1.5 h at room temperatutre. Blots were developed with Immobilon Western Chemiluminescent HRP Substrate (Millipore Corp.).

### Polyribosomal Gradients

Two grams of 15 day-old Arabidopsis thaliana seedlings were crushed with liquid nitrogen and homogenized in 2 ml of lysis buffer consisting of 200 mM Tris-HCl, 50 mM KAc, 25 mM MgAc, 2 mM EGTA 2, 2% PTE (10 Tridecyl Polyoxyethylene ether), 1% Nonidet P-40, now IGEPAL and 50 mg/ml cycloheximide. After the lysis, samples were centrifuged for 15 min at 12,000 g at 4°C. The supernatant was placed on 60% sucrose in gradient buffer containing 50 mM Tris-HCl, 20 mM KAc, 2 mM EGTA and 50 mg/ml cycloheximide and centrifuged in a Beckman 75Ti rotor at 50,000 rpm for 3 hours at 4°C. The pellet was dissolved in 400 µl of DEPC water, layered onto 20 to 60% sucrose continuous gradient and centrifuged in a Beckman SW40 rotor at 36,000 rpm for 2.5 h at 4°C. The gradient was fractionated with continuous monitoring of the absorbance at 260 nm.

### RNA isolation

For total RNA isolation, 100 mg of 15 day-old seedlings were crushed with liquid N_2_ and homogenized in Trizol ® Reagent (Invitrogen). Chloroform was added at 1/6 (v/v), the mixture was vortexed and then centrifuged at 12,000 *g* for 15 min at 4°C. The colorless upper aqueous phase was carefully collected and precipitated with 1 volume of isopropanol at room temperature. After centrifugation at 12,000 *g* for 20 min at 4°C, the RNA pellet was washed with 75% ethanol, the ethanol removed by an additional round of centrifugation and the RNA dissolved in 50 µl of diethyl pyrocarbonate (DEPC)-treated water.

For RNA isolation from sucrose gradient fractions, each fraction was treated with 0.5% sodium dodecyl sulfate (SDS) and 20 ug/ml proteinase K (Sigma), then extracted twice with an equal volume of phenol/chloroform/isoamyl alcohol [25∶24∶1 (v/v/v)] by vortexing. The mixture was centrifuged at 10,000 g for 5 min at 4°C. The aqueous phase was brought to 1.5 M with 10 M LiCl and an equal volume of cold isopropanol was added. The RNA was precipitated at −20°C for 1 h and centrifuged at 12,000 g for 15 min at 4°C. The pellet was washed with 70% ethanol and dissolved in 30 µl of DEPC-treated water.

Total or fractionated RNA was quantified at 260 nm and its integrity was analyzed on a denaturing agarose gel. The fractionated RNA was pooled in non-polyribosomal (NP) and polyribosomal (P) according to its electrophoretic pattern and the A_260_ profile of the sucrose gradient (Figure S3).

### Microarrays

Microarray slides were fabricated by the University of Arizona using the Qiagen-Operon Arabidospis Genome Array Ready Oligo Set (AROS V.3.0) containing 29,000 oligonucleotides (http://www.arizona.edu/microarray/). For pre-hybridization, the slides were re-hydrated with water vapor at 60°C, and fixed with two cycles of UV light (1200J). After boiling for two minutes at 92°C, slides were washed with 95% ethanol for one minute and pre-hybridzed in 5× SSC, 0.1% SDS and 1% BSA for one hour at 42°C. The slides were washed and dried for further hybridization. Ten µg of RNA were used for cDNA synthesis incorporating dUTP-Alexa_555_ or dUTP-Alexa_647_ by the First-Strand cDNA labeling kit (Invitrogen). Incorporation of the fluorophores (dUTP-CY5 and dUTP-CY3) was analyzed by the absorbance at 555 nm for Alexa_555_ and 650 nm for Alexa_647_. Equal quantities of labeled cDNA were hybridized using UniHyb (TeleChem International INC) to the arrays for 14 h at 42°C. Two replicate experiments were performed with RNA obtained from independent plant groups.

### Data acquisition and analysis of array images

Acquisition and quantification of array images was performed in ScanArray 4000 with its accompanying software ScanArray 4000 from Packard BioChips. All images were captured using 65% PMT gain, 70 to 75% laser power and 10 µm resolution at 50% scan rate. For each spot the Alexa_555_ and Alexa_647_ density mean value and the background mean value were calculated with software ArrayPro Analyzer from Media Cibernetics. Microarray data analysis was performed with free software genArise, developed in the Computing Unit of Cellular Physiology Institute of UNAM (http://www.ifc.unam.mx/genarise/). GenArise carry out a number of transformations: background correction, Lowess normalization, intensity filter, replicates analysis and selecting differentially expressed genes. The goal of genArise is to identify which of the genes show good evidence of being differentially expressed. The software identifies differentially expressed genes by calculating an intensity-dependent z-score. It uses a sliding window algorithm to calculate the mean and standard deviation within a window surrounding each data point, and define a z-score where z measures the number of standard deviations from the mean: z_i_ = [R_i_ – mean(R)]/sd(R), where z_i_ is the z-score for each element, R_i_ is the log-ratio for each element, and sd(R) is the standard deviation of the log-ratio. With this criterion, the elements with a z-score >1.5 standard deviations would be the significantly differentially expressed genes. All detailed protocols and data can be accessed online at http://www.ncbi.nlm.nih.gov/geo/query/acc.cgi?acc=GSE29386.

### Quantitative real time PCR (qRT-PCR)

Two micrograms of RNA were treated with RQ1 DNase (Promega Corp., Madison, WI, USA) and subjected to reverse transcription using M-MLV-RT (Invitrogen). The PCR was performed with gene-specific primers designed in the MacVector program (nucleotide sequences of gene-specific primers for SYBR Green analysis can be viewed in Table S1). The amplification was performed in a 7500 DNA analyzer (Applied Biosystems, Foster, CA) using the SYBR® Green PCR master mix (Applied Biosystems) for signal detection. To normalize the total amount of cDNAs present in reactions from total RNA source, the 18 S rRNA gene was co-amplified as control. The R value (relative expression of the gene) was calculated using the following equation: R = [(EgenX) ^(ΔCtgenX) ^(WT - mutant)]/[(EHkp) ^CtHkp (WT - mutant)], where E is the efficiency, genX is the gene in study and Hkp is the control gene (18 S). The concentrations of individual mRNAs were calculated by the 2^−ΔCt^ method described previously [14]. To analyze the distribution of selected mRNAs in polysomes separated by sucrose gradients the percentage of the RNA in NP and P fractions was determined according to the sum of the same RNA in all fractions as total.

### UTR analysis

The 5′ and 3′ UTR regions, based on the longest expressed sequence tags, were downloaded for mRNAs translationally affected in the *(iso)4E-1* mutant using The Arabidopsis Information Resource (TAIR10: 11/17/10 release). The sequences present in the Salk-Stanford-PGEC Arabidopsis ORF gene list [33] were used to determine their UTR length, presence and stability of secondary structures in the 5′UTR [41]; http://mfold.rit.albany.edu), and the presence of uORFs, uAUGs, IRES-like sequences and CPE elements by UTRScan (http://www.ba.itb.cnr.it/BIG/UTRScan) and RegRNA (http://regrna.mbc.nctu.edu.tw).

## Supporting Information

Figure S1 **Molecular characterization of eIF(iso)4E overexpressing** ***Arabidopsis thaliana*** **transgenic plants.**
(PDF)

Figure S2 **Protein structural modeling of eIF(iso)4E-GFP.**
(PDF)

Figure S3 **GFP localization in transgenic plants.**
(PDF)

Figure S4 **Sucrose gradient sedimentation profiles.**
(PDF)

Figure S5 **eIF(iso)4E expression patterns in different** ***Arabidopsis thaliana*** **tissues and developmental stages.**
(PDF)

Figure S6 **Classification of transcripts significantly changed at steady-state level in the** ***(iso)4E-1*** **mutant according to the biological process where they are involved.**
(PDF)

Figure S7 **Polyribosomal distribution of selected mRNAs in an eIF4E overexpressing transgenic line.**
(PDF)

Figure S8 **Increase in** ***PHO1*** **transcript level in response to phosphate deficiency.**
(PDF)

Figure S9 **Primary root length in three independent transgenic Arabidopsis lines overexpressing eIF(iso)4E.**
(PDF)

Table S1 **List of oligonucleotides used as primers for real time RT-PCR (qRT-PCR).**
(PDF)

Table S2 **List of mRNAs identified by microarray analysis that significantly decreased their levels in polyribosomes (P) and increased in non-polyribosomes (NP) in the** ***(iso)4E-1*** **mutant.**
(PDF)

Table S3 **List of mRNAs identified by microarray analysis that significantly increased their levels in polyribosomes (P) and decreased in non-polyribosomes (NP) significantly in the** ***(iso)4E-1*** **mutant.**
(PDF)
