# Supplementary material for: Translation Initiation Factor AteIF(iso)4E Is Involved in Selective mRNA Translation in Arabidopsis Thaliana Seedlings
Source: PLoS One. 2012 Feb 20;7(2):e31606. doi: 10.1371/journal.pone.0031606 (PMC3282757; doi:10.1371/journal.pone.0031606)
Supplement: Figure S1 — Molecular characterization of eIF(iso)4E overexpressing Arabidopsis thaliana transgenic plants. (PDF) [file pone.0031606.s001.pdf]

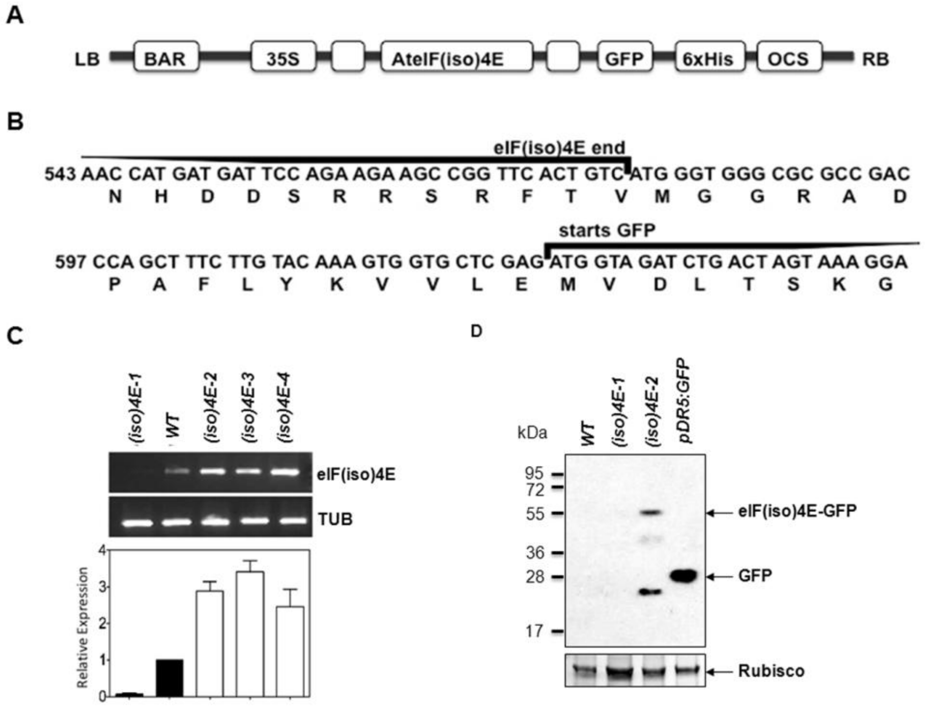

Supplementary Fig. S1: Molecular characterization of eIF(iso)4E overexpressing *Arabidopsis thaliana* transgenic plants. (**A)** Diagram of the construction bearing AteIF(iso)4E fused to GFP under the constitutive CaMV 35S promoter. LB, left border; BAR, glufosinate ammonium (BASTA) resistance gene; 6xHIS, histidine tag; OCS, octopine synthase transcription terminator; RB, right border. (**B)** Amino acid sequence of the above construct at the fusion site between eIF(iso)4E and GFP proteins. **(C)** Overexpression of the eIF(iso)4E transcript was measured by final point RT-PCR in three independent transgenic lines generated with the above construct: *(iso)4E-2*, *(iso)4E-3* and *(iso)4E-4*. The left panel indicates relative expression levels by densitometry in each transgenic line considering WT as 1. Tubulin (TUB) was used as internal control for RNA loading. The results represent three independent experiments. **(D)** Detection of proteins reacting against GFP antiserum. Total proteins were extracted from the lines used in this study as well as from a line expressing GFP alone under the auxin-responsive promoter DR5 (pDR5:GFP) as control. A 55 kDa band corresponding to the eIF(iso)4E-GFP fusion is seen only for *(iso)4E-2*, whereas a 28 kDa band corresponding to GFP is detected in pDR5:GFP. Two lower migrating bands also reacting with the antiserum are observed in the *(iso)4E-2* line suggesting that proteolysis may occur at some degree for the fusion protein.
