# Supplementary material for: Translation Initiation Factor AteIF(iso)4E Is Involved in Selective mRNA Translation in Arabidopsis Thaliana Seedlings
Source: PLoS One. 2012 Feb 20;7(2):e31606. doi: 10.1371/journal.pone.0031606 (PMC3282757; doi:10.1371/journal.pone.0031606)
Supplement: Figure S2 — Protein structural modeling of eIF(iso)4E-GFP. (PDF) [file pone.0031606.s002.pdf]

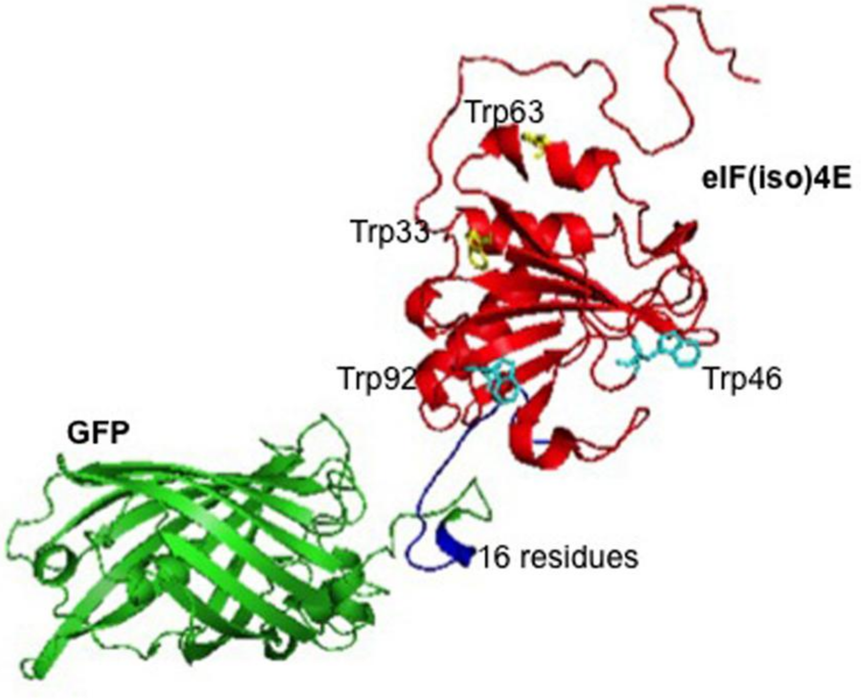

Supplementary Fig. S2 Protein structural modeling of eIF(iso)4E-GFP. The structure was modeled using the SAM-T08 server. Frontal views are shown with eIF(iso)4E in red, GFP in green and the amino acid sequence between the two fused proteins in blue. The residues Trp46, Trp92 involved in cap-binding are shown in cyan, and Trp33, Trp63 involved in eIF(iso)4G interaction in yellow. Amino acid numbering is from *Arabidopsis thaliana* eIF(iso)4E.
