# Supplementary material for: Translation Initiation Factor AteIF(iso)4E Is Involved in Selective mRNA Translation in Arabidopsis Thaliana Seedlings
Source: PLoS One. 2012 Feb 20;7(2):e31606. doi: 10.1371/journal.pone.0031606 (PMC3282757; doi:10.1371/journal.pone.0031606)
Supplement: Figure S3 — GFP localization in transgenic plants. (PDF) [file pone.0031606.s003.pdf]

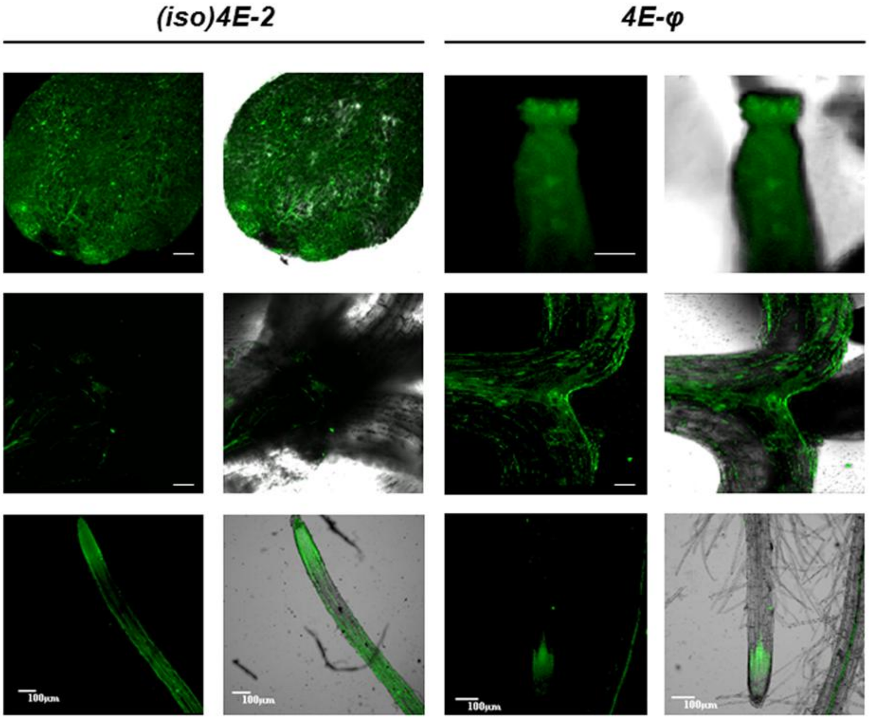

Supplemetary Fig. S3 The GFP fusion versions of eIF(iso)4E [*(iso)4E-2*] and eIF4E [*4E-φ*] expressed under the CaMV 35S promoter were observed in the tissues of 15 days-old seedlings, such as leaves, roots and aerial meristem. In addition, eIF4E-GFP detection is shown in pistil (upper panel). Images compiled from Z confocal microscopy are shown with white bars indicating 100 µm.
