# Supplementary material for: Translation Initiation Factor AteIF(iso)4E Is Involved in Selective mRNA Translation in Arabidopsis Thaliana Seedlings
Source: PLoS One. 2012 Feb 20;7(2):e31606. doi: 10.1371/journal.pone.0031606 (PMC3282757; doi:10.1371/journal.pone.0031606)
Supplement: Figure S4 — Sucrose gradient sedimentation profiles. (PDF) [file pone.0031606.s004.pdf]

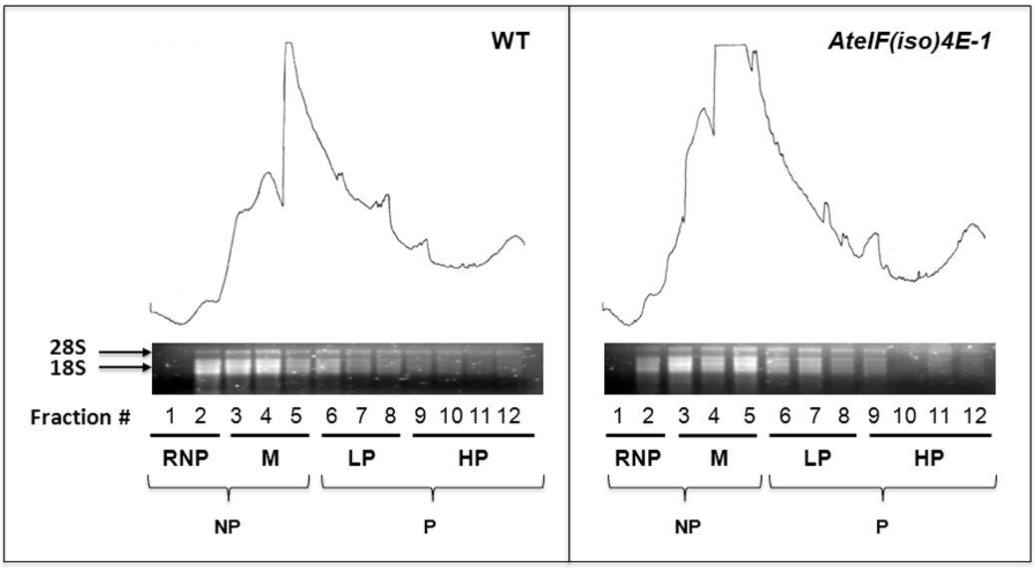

Supplementary Fig. S4 Sucrose gradient sedimentation profiles. Ribosomal fractions from *Arabidopsis thaliana* wild type (WT) and mutant [*(iso)4E-1*] 15 day-old whole seedlings were fractionated on 20-60% sucrose gradients. One milliliter fractions were collected with continuous monitoring of Absorbance at 260 nm (solid line). Each fraction was analyzed by agarose electrophoresis to detect the ribosomal RNA 28S and 18S integrity. Fractions 1-5 were considered as non-polyribosomal (NP) and 6-12 as polyribosomal (P). For some experiments, NP were pooled as free RNP (fractions 1, 2) and monosomes (M; fractions 3-5), whereas polysomes were pooled as low polysomes (LP; fractions 6-8) and high polysomes (HP; fractions 9-12).
