# Supplementary material for: Translation Initiation Factor AteIF(iso)4E Is Involved in Selective mRNA Translation in Arabidopsis Thaliana Seedlings
Source: PLoS One. 2012 Feb 20;7(2):e31606. doi: 10.1371/journal.pone.0031606 (PMC3282757; doi:10.1371/journal.pone.0031606)
Supplement: Figure S5 — eIF(iso)4E expression patterns in different Arabidopsis thaliana tissues and developmental stages. (PDF) [file pone.0031606.s005.pdf]

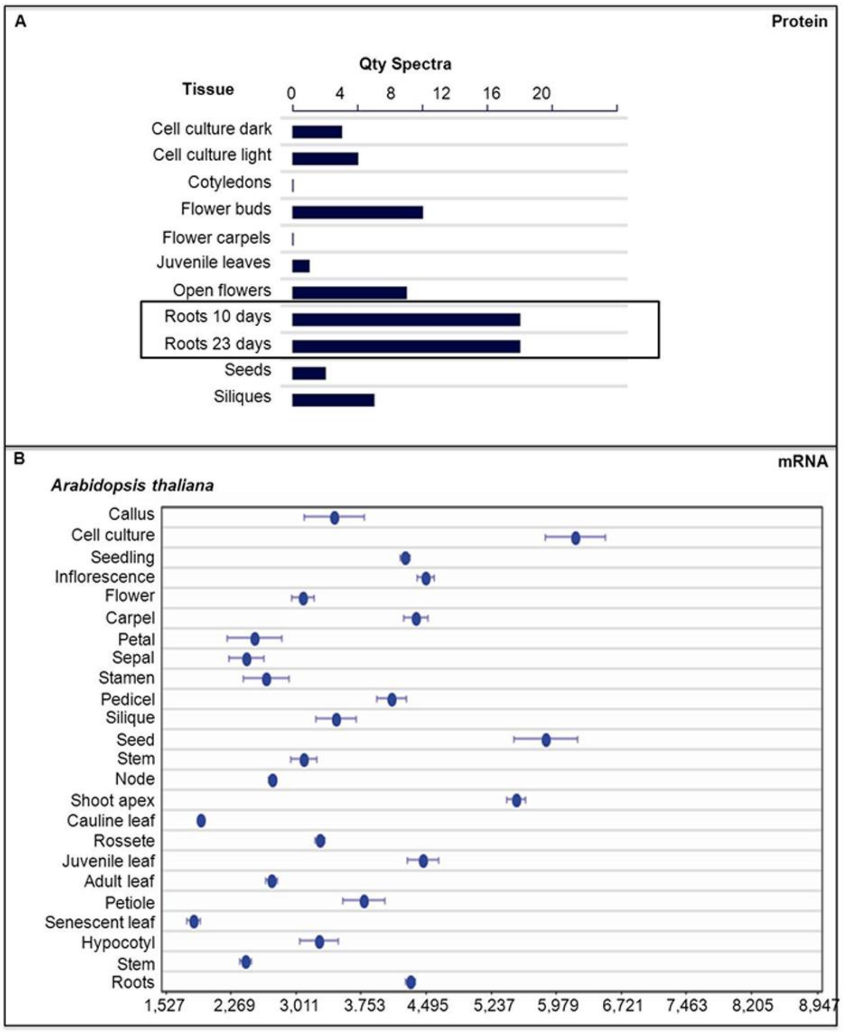

Supplementary Fig. S5 eIF(iso)4E expression patterns in different *Arabidopsis thaliana* tissues and developmental stages. **(A)** According to the AtProteome site (Baerenfaller, et al., 2008), the eIF(iso)4E protein is more abundant in roots of 10 and 23 days old plants. **(B)** According to the microarray data available at Genevestigator (Hruz et al., 2008), the *eIF(iso)4E* mRNA is expressed in most tissues, with greater abundance cell culture, mature seed and shoot apex. Noticeably, the mRNA expression pattern does not overlap with the protein abundance of this protein.
