# Supplementary material for: Translation Initiation Factor AteIF(iso)4E Is Involved in Selective mRNA Translation in Arabidopsis Thaliana Seedlings
Source: PLoS One. 2012 Feb 20;7(2):e31606. doi: 10.1371/journal.pone.0031606 (PMC3282757; doi:10.1371/journal.pone.0031606)
Supplement: Figure S6 — Classification of transcripts significantly changed at steady-state level in the (iso)4E-1 mutant according to the biological process where they are involved. (PDF) [file pone.0031606.s006.pdf]

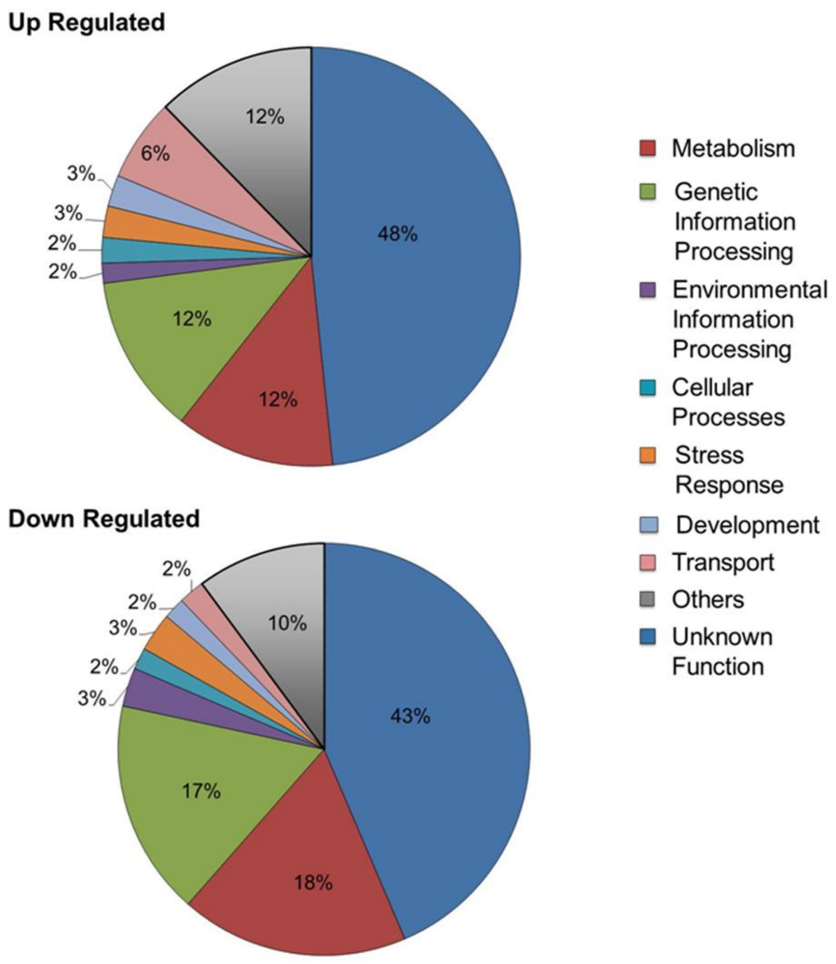

Supplementary Fig. S6 Classification of transcripts significantly changed at steady-state level in the *(iso)4E-1* mutant according to the biological process where they are involved. Total RNA extracted from 15 day-old whole seedlings was used for a Microarray analysis to detect genes significantly changed in their transcription in the *(iso)4E-1* mutant. The threshold for significant changes was a z-score of 2 either up or down. The analysis revealed 496 genes up regulated in *(iso)4E-1* mutant plants, whereas 296 were repressed. According to the reported gene onthology (GO) for Biological Process, a great proportion of genes with unknown function appeared changed, followed by a major group of genes related to metabolism or genetic information processing.
