# Supplementary material for: Translation Initiation Factor AteIF(iso)4E Is Involved in Selective mRNA Translation in Arabidopsis Thaliana Seedlings
Source: PLoS One. 2012 Feb 20;7(2):e31606. doi: 10.1371/journal.pone.0031606 (PMC3282757; doi:10.1371/journal.pone.0031606)
Supplement: Figure S7 — Polyribosomal distribution of selected mRNAs in an eIF4E overexpressing transgenic line. (PDF) [file pone.0031606.s007.pdf]

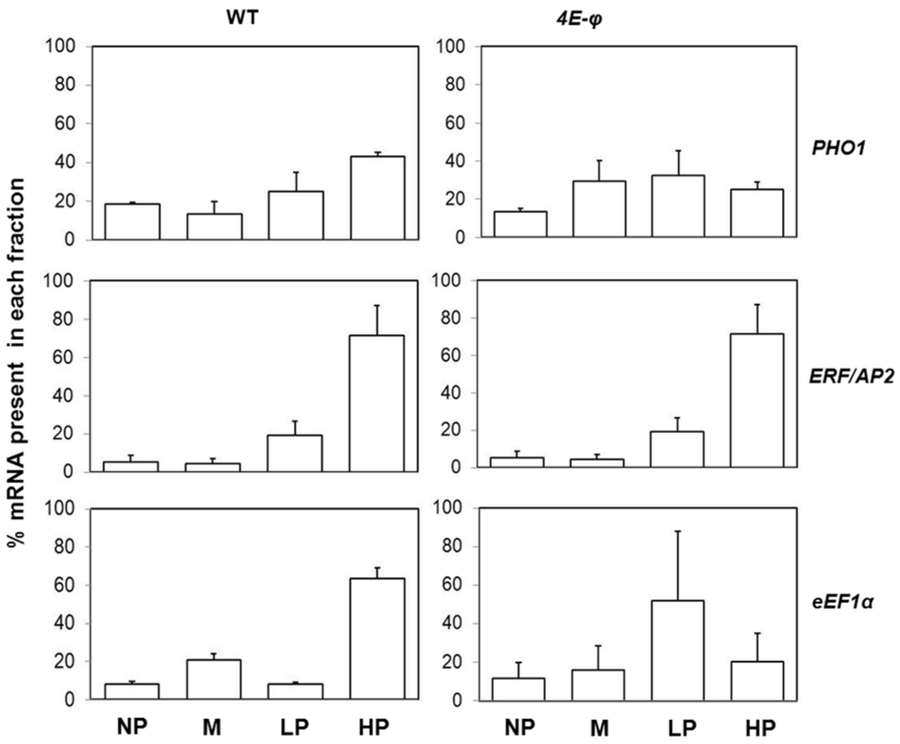

Supplementary Fig. S7 Polyribosomal distribution of selected mRNAs in an eIF4E overexpressing [*4E-φ*] transgenic line. Ribosomal fractionation on sucrose density gradients (20-60%) was performed for *Arabidopsis thaliana* wild type (WT) and mutant [*4E-φ*] 15 day-old whole seedlings. The percentage of mRNA found in free RNP (NP), monosomes (M), low polyribosomes (LP) and high polyribosomes (HP), collected according to Supplementary figure S4, was calculated by qRT-PCR as described in Materials and Methods. Translation elongation factor *eIF1α* was used as control mRNA. The data represent average of two independent replicates and bars indicate the standard error.
