# Supplementary material for: Translation Initiation Factor AteIF(iso)4E Is Involved in Selective mRNA Translation in Arabidopsis Thaliana Seedlings
Source: PLoS One. 2012 Feb 20;7(2):e31606. doi: 10.1371/journal.pone.0031606 (PMC3282757; doi:10.1371/journal.pone.0031606)
Supplement: Figure S8 — Increase in PHO1 transcript level in response to phosphate deficiency. (PDF) [file pone.0031606.s008.pdf]

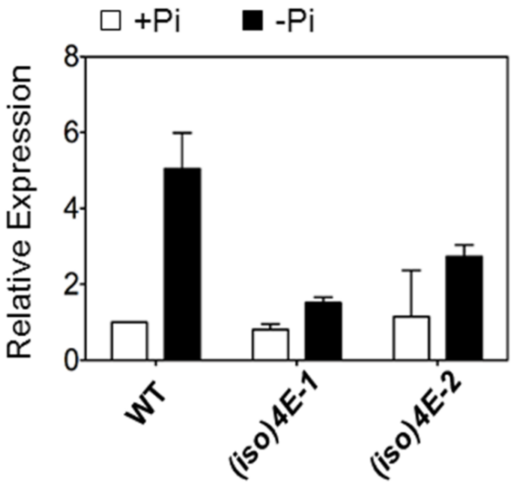

Supplementary Fig. S8 Increase in *PHO1* transcript level in response to phosphate deficiency. Plants were grown for 15 days in Gamborg's B5 liquid medium and transferred to Hoagland medium containing phosphate (+Pi, white columns) or without phosphate (-Pi, black columns) for 10 days. Total RNA was obtained, DNase treated and assayed by qRT-PCR using as internal control the 18S rRNA gene. Data are shown as the mean of three independent experiments with three replicates each. Bars represent standard error.
