# Supplementary material for: Translation Initiation Factor AteIF(iso)4E Is Involved in Selective mRNA Translation in Arabidopsis Thaliana Seedlings
Source: PLoS One. 2012 Feb 20;7(2):e31606. doi: 10.1371/journal.pone.0031606 (PMC3282757; doi:10.1371/journal.pone.0031606)
Supplement: Figure S9 — Primary root length in three independent transgenic Arabidopsis lines overexpressing eIF(iso)4E. (PDF) [file pone.0031606.s009.pdf]

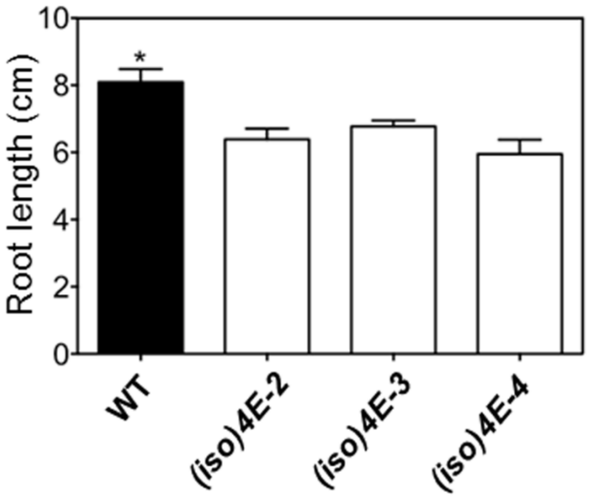

Supplementary Fig. S9 Primary root length in three independent transgenic Arabidopsis lines overexpressing eIF(iso)4E. Wild type (WT) and transgenic *(iso)4E-2*, *(iso)4E-3* and *(iso)4E-4* plants were grown for 21 days on Hoagland solid medium placed vertically in growth chamber. Each plant was carefully removed from the medium and measured for primary root length as described in Materials and methods. Data are shown as the mean of 5 independent experiments with 5 plants per experiment. Bars indicate the standard error. One way ANOVA with Tukey's Multiple Comparison Test using a p value < 0.05 was used for statistical analysis. Significant differences between WT and all three transgenic lines overexpressing eIF(iso)4E are indicated by asterisk.
