# Supplementary material for: Translation Initiation Factor AteIF(iso)4E Is Involved in Selective mRNA Translation in Arabidopsis Thaliana Seedlings
Source: PLoS One. 2012 Feb 20;7(2):e31606. doi: 10.1371/journal.pone.0031606 (PMC3282757; doi:10.1371/journal.pone.0031606)
Supplement: Table S1 — List of oligonucleotides used as primers for real time RT-PCR (qRT-PCR). (PDF) [file pone.0031606.s010.pdf]

Table S1. Oligonucleotide sequences used in real time PCR experiments (qRT-PCR)

Gene ID	Other Name	Forward	Reverse	Product length (bp)
At1g21630		CACAACAGGGTTCATTGAGCCAG	AGGTGGACCAGAAGCCGCAC	104
At1g64580		TTCTGATTCATTGTTTCTGCCGATG	CGTTGAGGAGAGAGCCAAGAGTGAC	73
At1g68670		TCGGAAGGAGTTATCTGGTACGACG	AAGACAGGACCACCACAAACACTTG	84
At1g68800	BRC2	AGGCGAGCAAGACGATTGAATG	CCTTTCCTCCTCCTTCCGATGC	96
At1g78240	TSD2	CCATTCCCGACTTACCCGAGAAC	TCCCTCTGGACGAAGCAATCG	138
At2g02860	SUC3	CGCAATCGTTATTCCCCAGATG	AAGCAGCAACAGACGCCAAAAC	107
At2g03710	AGL3	CCTTCTCTACCCAGACAGCGTGG	CACCCCATCCAGCAACTCCC	80
At2g17630		AACCCAACACCATCAACCTCACC	AATGCGGCTGGACCTGCG	121
At2g27940		TTCCGCCACTTCGCAGACAG	CCTGAGAATCAAGACCCCTCCG	148
At2g28600		ATGGCGAAAGGAGACGATAATGTG	ATGGCGGCGATACGAGCAG	107
At2g30260	U2B"	CCCAACGACCAAACACGGC	TCGGCTGGAACGAAGGCG	84
At3g23430	PHO1	CCAAACACAGCGTTGCGTTACC	GAGGCTCGGAGGATGAGGTGG	101
At3g55580	RCC1	CAGTCTGACAACATTGACCTCTCGG	GTATGACGACCACCAGCAGCAAC	95
At3g57600	ERF/AP2	TGGAAGAAGGGTCCTGCTCGG	CAAGTCCTTTGCCTGACTCCACG	80
At4g06746	RAP2.9	GTTCATACAAAACCGCCGTTGC	TGAGTCTCGCCGAAGGACCAC	81
At4g18720	TEF-R	AGCGAGACAAAGTGCGTGAGATTC	CCACAGATACAGCCACAACCCAAG	123
At4g20340	TFIIE	AGCCGTTTGTCAAGCTGGTGAG	TCTAACCCATTGCCGCCTCG	149
At4g33250	EIF3K	CCAGACTTCAGCCTTTGCCTCTTC	CCAGAACTGTTGGAACCTCCCAG	117
At5g01840	OFP1	GGAACCGTCTTCGTCACCGAG	ACCTGTTTCTGTGGTAGTGCTGTCG	80
At5g15630	COBL4	CGGCAGATGGCTATGTGGCTAC	TGTCCAACCTAATGTCCAACCAGG	89
At5g15850	COL1	ATGCTGCCAATCGTCTTGCTTC	TGCGGAATGAATCTCTGAATCACAG	137
At5g61430	NAC5	CCTGCTTCTCCAACCAAACGG	AATCCTGTGAAAAATGTCGGCTTG	101
