# Supplementary material for: Translation Initiation Factor AteIF(iso)4E Is Involved in Selective mRNA Translation in Arabidopsis Thaliana Seedlings
Source: PLoS One. 2012 Feb 20;7(2):e31606. doi: 10.1371/journal.pone.0031606 (PMC3282757; doi:10.1371/journal.pone.0031606)
Supplement: Table S2 — List of mRNAs identified by microarray analysis that significantly decreased their levels in polyribosomes (P) and increased in non-polyribosomes (NP) in the (iso)4E-1 mutant. (PDF) [file pone.0031606.s011.pdf]

Table S2. mRNAs shifted from polyribosomes towards non-polyribosomes in *(iso)4E-1* 15 day-old seedlings.

**Gene ID**	**Fraction**[a]	**Zscore**[b]	**Function/Gene name**[c]	**Biological Process**[c]	**Cellular Component**[c]	**Plant structure**[d]
At1g52940	P	-2.856.419	Purple acid phosphatase 5 (PAP5)	Unknown	Unknown	Inflorescence
	NP	4.008.580				
At5g01840	P	-2.832.152	Member of the plant-specific ovate family protein (OFP1)	Regulation of unidimensional cell growth	Cytoskeleton, nucleolus	Root
	NP	3.645.499				
At1g11300	P	-2.760.778	Protein Serine/Threonine kinase	Protein phosphorylation	Endomembrane system	Inflorescence
	NP	1.536.855				
At4g20340	P	-2.758.056	Transcription initiation factor TFIIE, alpha subunit	RNA polymerase II directed transcription	Endomembrane system	Unknown
	NP	2.952.168				
At4g02465	P	-2.744.616	Unknown protein	Unknown	Unknown	Unknown
	NP	2.094.202				
At4g24490	P	-2.679.040	RAB geranylgeranyl transferase alpha subunit 1 (RGTA1)	Response to cadmium ion	Unknown	Root
	NP	2.840.810				
At1g21630	P	-2.633.983	Calcium ion binding, EF hand family protein	Unknown	Cytosol, plasma membrane	Rosette
	NP	3.550.155				
At3g55580	P	-2.579.383	Regulator of chromosome condensation, (RCC1) family protein	Unknown	Unknown	Root
	NP	2.510.298				
At2g30260	P	-2.567.622	component of the U2 snRNP complex (U2B")	Cis assembly of pre-catalytic spliceosome	Cajal body, cytoplasm, nucleoplasm	Root
	NP	2.542.680				
At5g22610	P	-2.520.831	F-box/RNI-like/FBD-like domains-containing protein	Unknown	Unknown	Unknown
	NP	2.157.099				
At5g48740	P	-2.490.928	Leucine-rich repeat protein kinase family protein	Protein phosphorylation	Endomembrane system	Root
	NP	2.051.598				
At1g50220	P	-2.448.991	Unknown protein	Unknown	Unknown	Unknown
	NP	2.508.035				
At1g12570	P	-2.394.780	Glucose-methanol-choline (GMC)	Alcohol metabolic	Endomembrane	Rosette

	NP	1.757.340	oxidoreductase family protein	process, electron transport	system	
At1g07280	P	-2.372.525	Tetratricopeptide repeat (TPR)-like superfamily protein	Unknown	Chloroplast	Rosette
	NP	1.986.108				
At1g29700	P	-2.333.855	Metallo-hydrolase/oxidoreductase superfamily protein	Unknown	Chloroplast	Rosette
	NP	2.782.488				
At3g23430	P	-2.324.267	mutant is deficient in the transfer of phosphate from root epidermal and cortical cells to the xylem (PHO1)	cellular response to phosphate starvation, phosphate transport	Integral to the membrane	Root
	NP	1.583.184				
At5g02800	P	-2.308.138	Protein kinase (Serine/Threonine) family protein	Protein phosphorylation	Unknown	Rosette
	NP	3.191.886				
At5g11280	P	-2.240.252	Unknown protein	Unknown	Unknown	Root
	NP	2.047.710				
At5g50590	P	-2.222.029	Encodes a putative hydroxysteroid dehydrogenase (HSD4)	Metabolic process, oxidation-reduction process	Endomembrane system	Root
	NP	2.213.526				
At1g05550	P	-2.183.651	Protein of unknown function (DUF295)	Unknown	Unknown	Rosette
	NP	2.213.054				
At1g68480	P	-2.177.591	Encodes a putative zinc finger transcription factor (JAG)	Abaxial cell fate specification, flower development	Nucleus	Inflorescence
	NP	1.515.466				
At4g02950	P	-2.159.562	Ubiquitin family protein	Protein modification	Unknown	Inflorescence
	NP	2.802.140				
At2g17630	P	-2.157.554	Pyridoxal phosphate (PLP)-dependent transferases superfamily protein	Response to cadmium ion	Chloroplast	Root
	NP	2.505.533				
At5g48140	P	-2.086.828	Pectin lyase-like superfamily protein	Carbohydrate metabolic process	Endomembrane system	Inflorescence
	NP	2.921.912				
At1g22885	P	-2.075.976	Unknown protein	Unknown	Endomembrane system	Rosette
	NP	2.036.507				
At5g19630	P	-2.055.451	alpha/beta-Hydrolases superfamily protein	Unknown	Unknown	Root
	NP	1.908.328				
At1g64580	NP	-2.034.944	Pentatricopeptide repeat (PPR)	Unknown	Unknown	Rosette

	P	1.578.590	superfamily protein			
At1g64330	P	-2.032.722	Myosin heavy chain-related	Unknown	Vacuolar membrane, vacuole	Root
	NP	2.169.154				
At2g07680	P	-2.008.768	Multidrug resistance-associated protein 11 (MRP11)	Transport	Plasma membrane	Root
	NP	1.673.276				
At3g44785	P	-2.004.434	Zinc finger C-x8-C-x5-C-x3-H type family protein	Unknown	Nucleus	Unknown
	NP	1.593.863				
At2g20590	P	-1.982.197	Reticulon family protein	Unknown	Endoplasmic reticulum	Unknown
	NP	2.689.850				
At3g22250	P	-1.978.017	UDP-glycosyl transferase superfamily protein	Metabolic process	Unknown	Inflorescence
	NP	1.686.238				
At1g21020	P	-1.975.634	Transposable element gene, similar to Ulp1 protease family protein	Unknown	Unknown	Inflorescence
	NP	3.302.254				
At1g04650	P	-1.970.616	Unknown protein	Unknown	Unknown	Inflorescence
	NP	3.135.925				
At1g03100	P	-1.966.563	Pentatricopeptide repeat (PPR) superfamily protein	Unknown	Unknown	Inflorescence
	NP	1.818.271				
At2g02860	P	-1.948.161	Sucrose transporter in sieve elements and sink tissues. Sucrose transporter 3 (SUC3)	Response to wounding, sucrose transport	Plasma membrane	Root
	NP	1.612.801				
At1g07390	P	-1.924.124	Receptor like protein 1 (RLP1)	Signal transduction	Unknown	Inflorescence
	NP	2.541.153				
At1g08610	P	-1.879.840	Pentatricopeptide repeat (PPR) superfamily protein	Unknown	Unknown	Rosette
	NP	1.531.292				
At4g09280	P	-1.873.907	Transposable element gene; similar to Ulp1 protease family protein	Unknown	Unknown	Unknown
	NP	2.515.281				
At5g44410	P	-1.873.764	FAD-binding Berberine family protein	Electron transport, oxidation-reduction process	Endomembrane system	Root
	NP	3.821.529				
At3g19130	P	-1.869.365	RNA-binding protein 47B (RBP47B)	Unknown	Unknown	Root
	NP	1.565.215				

At2g24762	P	-1.853.583	Member of the GDU (glutamine dumper) family proteins involved in amino acid export (GDU4)	Regulation of amino acid export	Unknown	Root
	NP	1.771.616				
At4g04220	P	-1.839.983	Receptor like protein 46 (RLP46)	Defense response, signal transduction	Endomembrane system	Rosette
	NP	1.925.787				
At2g20270	P	-1.838.934	Thioredoxin superfamily protein	Electron transport, cell redox homeostasis	Chloroplast	Rosette
	NP	1.784.900				
At1g64185	P	-1.838.622	Lactoylglutathione lyase / glyoxalase I family protein	Metabolic process	Unknown	Inflorescence
	NP	2.239.101				
At3g49000	P	-1.818.054	RNA polymerase III subunit RPC82 family protein	Transcription	Endomembrane system	Inflorescence
	NP	1.509.432				
At4g31310	P	-1.800.673	AIG2-like (avirulence induced gene) family protein	Unknown	Unknown	Rosette
	NP	1.910.589				
At1g63290	P	-1.782.392	Aldolase-type TIM barrel family protein	Carbohydrate metabolic process	Endomembrane system	Seedling
	NP	1.976.152				
At3g20950	P	-1.774.474	Cytochrome P450, family 705, subfamily A, polypeptide 32 (CYP705A32)	Electron transport	Endomembrane system	Inflorescence
	NP	1.859.443				
At4g01925	P	-1.763.523	Cysteine/Histidine-rich C1 domain family protein	Unknown	Unknown	Unknown
	NP	1.642.617				
At5g20510	P	-1.762.377	Member of the Alfin-Like family (AL5), binds to H3K4 di or trimethylated	Regulation of transcription	Nucleus	Rosette
	NP	1.598.593				
At5g23300	P	-1.737.886	Dihydroorotate dehydrogenase, catalyses the fourth step of pyrimidine biosynthesis (PYRD)	pyrimidine ribonucleotide biosynthetic process	Mitochondrion, plastid	Inflorescence
	NP	2.508.113				
At4g26190	P	-1.721.274	Haloacid dehalogenase-like hydrolase (HAD) superfamily protein	Unknown	Unknown	Inflorescence
	NP	3.194.047				
At4g31970	P	-1.716.411	Cytochrome P450, family 82, subfamily C, polypeptide 2 (CYP82C2)	Electron transport, oxidation-reduction process	Endomembrane system	Root
	NP	2.102.280				
At4g08455	P	-1.712.692	BTB/POZ domain-containing protein	Unknown	Unknown	Unknown
	NP	2.225.747				

At1g21310	P	-1.704.186	Encodes extensin 3 (EXT3)	Plant-type cell wall organization	Endomembrane system	Root
	NP	1.904.038				
At4g01570	P	-1.703.545	Tetratricopeptide repeat (TPR)-like superfamily protein	Unknown	Unknown	Root
	NP	2.020.663				
At2g40560	P	-1.703.451	Protein kinase (Serine/Threonine) superfamily protein	Protein phosphorylation	Unknown	Unknown
	NP	3.181.962				
At3g13175	P	-1.698.878	Unknown protein	Unknown	Endomembrane system	Inflorescence
	NP	1.783.530				
At1g66040	P	-1.694.406	Predicted as a protein with N-terminal PHD domain and two RING domains surrounding an SRA domain (ORTH/VIM)	Unknown	Unknown	Root
	NP	3.165.143				
At2g42350	P	-1.669.320	RING/U-box superfamily protein	Unknown	Unknown	Root
	NP	2.696.397				
At2g22750	P	-1.660.457	basic helix-loop-helix (bHLH) DNA-binding superfamily protein	Regulation of transcription	Nucleus	Root
	NP	3.586.507				
At3g04950	P	-1.656.873	Unknown protein	Unknown	Unknown	Inflorescence
	NP	1.557.073				
At1g21990	P	-1.652.637	F-box/RNI-like/FBD-like domains-containing protein	Unknown	Unknown	Unknown
	NP	1.748.356				
At5g15630	P	-1.642.701	Member of the COBRA family, similar to phytochelatin synthetase	Secondary cell wall biogenesis	Plasma and vacuolar membrane	Seedling
	NP	2.859.030				
At1g53290	P	-1.634.323	Galactosyltransferase family protein	Protein glycosylation	Membrane	Seedling
	NP	3.033.373				
At1g17480	P	-1.621.719	IQ-domain (IQD7), calmodulin binding	Unknown	Unknown	Root
	NP	1.826.055				
At1g19020	P	-1.613.449	Unknown protein	Response to oxidative stress	Unknown	Rosette
	NP	1.565.207				
At3g14560	P	-1.607.103	Unknown protein	Unknown	Unknown	Inflorescence
	NP	1.868.781				
At3g57990	P	-1.598.408	Unknown protein	Unknown	Unknown	Rosette

	NP	1.746.236				
At1g74640	P	-1.586.131	Alpha/beta-Hydrolases superfamily protein	Unknown	Chloroplast	Rosette
	NP	2.856.396				
At3g55920	P	-1.581.875	Cyclophilin-like peptidyl-prolyl cis-trans isomerase family protein	Protein folding	Plasma membrane	Inflorescence
	NP	1.984.927				
At4g14805	P	-1.578.871	Bifunctional inhibitor/lipid-transfer protein/seed storage 2S albumin superfamily protein	Unknown	Anchored to membrane	Unknown
	NP	1.619.084				
At3g63470	P	-1.570.200	Serine carboxypeptidase (SCPL40)	Proteolysis	Endomembrane system	Root
	NP	2.485.057				
At1g48520	P	-1.569.089	Glu-tRNA(Gln) amidotransferase subunit B (GATB)	glutamyl-tRNA aminoacylation, translation	Chloroplast	Rosette
	NP	3.277.840				
At1g80910	P	-1.552.809	Protein of unknown function (DUF1712)	Unknown	Unknown	Root
	NP	1.707.097				
At1g78240	P	-1.550.465	Tumorous shoot development 2 (TSD2)	Homogalacturonan biosynthetic process, root and shoot development	Golgi apparatus	Root
	NP	1.604.572				
At1g63380	P	-1.548.097	NAD(P)-binding Rossmann-fold superfamily protein	metabolic process, oxidation-reduction	Unknown	Rosette
	NP	2.528.263				
At2g24360	P	-1.534.877	Protein kinase (Serine/Threonine/Tyrosine) superfamily	Protein phosphorylation	Plasma Membrane	Inflorescence
	NP	1.709.761				

[a] Polyribosomes (P) and non-polyribosomes (NP) were obtained by sucrose gradient (20-60%) fractionation as described in Materials and methods and shown in Figure S4.

[b] The GeneArise software identified differentially distributed mRNAs in *AteIF(iso)4E-1* fractions considering a z-score > 1.5 standard deviations.

[c] Data were taken from The Arabidopsis Resource Center (TAIR).

[d] The plant structure reported with the higher mRNA level was considered according to the AtGenexpress Visualization Tool (Schmid, M., Davison, T.S., Henz, S.R., et al., 2005, A gene expression map of Arabidopsis thaliana development, Nature Genetics 37: 501-506).
