# Supplementary material for: Translation Initiation Factor AteIF(iso)4E Is Involved in Selective mRNA Translation in Arabidopsis Thaliana Seedlings
Source: PLoS One. 2012 Feb 20;7(2):e31606. doi: 10.1371/journal.pone.0031606 (PMC3282757; doi:10.1371/journal.pone.0031606)
Supplement: Table S3 — List of mRNAs identified by microarray analysis that significantly increased their levels in polyribosomes (P) and decreased in non-polyribosomes (NP) significantly in the (iso)4E-1 mutant. (PDF) [file pone.0031606.s012.pdf]

Table S3. mRNAs shifted from non-polyribosomes towards polyribosomes in the *(iso)4E-1* 15 day-old seedlings.

Gene ID	Fraction[a]	Zscore[b]	Function/Gene name[c]	Biological Process[c]	Cellular Component[c]	Plant structure[d]
At4g18720	NP	-2.673.546	Transcription factor IIS protein	Transcription	Nucleus	Inflorescence, Seed
	P	3.161.070				
At1g51000	NP	-2.230.721	Unknown protein	Unknown	Unknown	Seed
	P	3.047.951				
At3g30160	NP	-2.142.414	Unknown protein	Unknown	Endomembrane system	Inflorescence
	P	2.982.585				
At3g47830	NP	-1.545.177	DNA glycosylase superfamily protein	DNA repair, base-excision repair	Unknown	Unknown
	P	2.944.518				
At2g17080	NP	-2.098.657	Arabidopsis protein of unknown function (DUF241)	Unknown	Chloroplast	Root
	P	2.925.778				
At5g56740	NP	-2.538.741	H4 Histone acetyltransferase (HAC7)	Chromatin modification, histone acetylation	Nucleus	Inflorescence
	P	2.816.778				
At2g05710	NP	-3.258.442	Aconitase (ACO3)	Metabolic process, abscisic acid, cadmium ion, oxidative stress, salt stress	Cell wall, chloroplast, cytosol, mitochondrion, plasma membrane	Inflorescence
	P	2.635.277				
At4g33250	NP	-1.787.014	Eukaryotic translation initiation factor 3K (eIF3K)	Regulation of translational initiation	Intracellular	Inflorescence
	P	2.587.417				
At3g62630	NP	-1.902.524	Protein of unknown function (DUF1645)	Unknown	Unknown	Inflorescence
	P	2.341.149				
At5g61430	P	-1.841.136	NAC domain containing protein (NAC5)	Transcription regulation	Unknown	Inflorescence
	NP	2.314.377				
At4g09740	NP	-1.588.788	glycosyl hydrolase 9B14 (GH9B14)	Carbohydrate metabolism	Endomembrane system	Inflorescence
	P	2.277.257				

At5g46660	NP	-1.939.306	Protein kinase C-like zinc finger protein	Unknown	Unknown	Inflorescence
	P	2.230.663				
At1g66310	NP	-1.571.516	F-box/RNI-like/FBD-like domains-containing protein	Unknown	Unknown	Inflorescence
	P	2.148.245				
At5g04680	NP	-2.223.525	Ankyrin repeat family protein	Unknown	Unknown	Inflorescence, Rosette
	P	2.137.576				
At3g16750	NP	-1.675.123	Unknown protein	Unknown	Unknown	Inflorescence, Rosette
	P	2.104.560				
At4g06746	NP	-1.785.573	Encodes a member of the DREB subfamily A-5 of ERF/AP2 transcription factor family (RAP2.9)	Transcription regulation	Nucleus	Unknown
	P	2.075.933				
At5g46870	NP	-1.981.270	RNA-binding (RRM/RBD/RNP motifs) family protein	Oxidation-reduction	Unknown	Unknown
	P	2.074.465				
At2g24255	NP	-1.876.917	Protein of unknown function (DUF295)	Unknown	Unknown	Inflorescence
	P	2.067.121				
At1g77540	NP	-1.680.218	H3/H4 Histone acetyltransferase	Unknown	Peroxisome	Inflorescence
	P	2.035.870				
At2g16587	NP	-2.105.544	Expressed protein (Obsolete)			
	P	2.001.793				
At1g74320	NP	-1.605.135	Choline kinase	Unknown	Unknown	Inflorescence
	P	1.995.300				
At2g40980	NP	-1.934.189	Protein kinase superfamily protein	Unknown	Plasma membrane	Inflorescence
	P	1.985.620				
At5g42990	NP	-1.947.571	Ubiquitin-conjugating enzyme 18 (UBC18)	Ubiquitin-dependent protein catabolic process	Unknown	Seed
	P	1.962.867				
At5g46320	NP	-1.636.239	MADS-box family	Unknown	Unknown	Inflorescence,

	P	1.944.284	protein			Seed
At5g37000	NP	-1.716.726	Exostosin family protein	Unknown	Endomembrane system	Unknown
	P	1.923.488				
At5g58950	NP	-1.552.318	Protein kinase (Serine/Threonine/Tyrosine) superfamily protein	Protein phosphorylation	Cytosol, plasma membrane	Inflorescence
	P	1.906.344				
At2g28430	NP	-1.630.853	Unknown protein	Unknown	Unknown	Rosette
	P	1.853.080				
At5g34895	NP	-1.842.650	Transposable element gene; similar to heat shock protein binding	Unknown	Unknown	Seed
	P	1.847.307				
At2g28600	NP	-2.087.557	P-loop containing nucleoside triphosphate hydrolases superfamily protein	Unknown	Unknown	Inflorescence
	P	1.827.262				
At2g27940	NP	-1.980.991	RING/U-box superfamily protein	Unknown	Unknown	Seed
	P	1.811.982				
At1g68670	NP	-1.831.687	Myb-like transcription factor family protein	Regulation of transcription	Nucleus	Rosette
	P	1.798.796				
At4g16850	NP	-2.202.095	Unknown protein	Unknown	Unknown	Root
	P	1.786.194				
At1g34070	NP	-1.809.017	Retrotransposon gag protein	Unknown	Unknown	Seed
	P	1.781.528				
At5g15850	NP	-2.044.311	Transcription factor, constans-like 1 (COL1)	Regulation of flower development	Nucleus	Rosette
	P	1.716.399				
At4g36420	NP	-1.578.407	Ribosomal protein L12 family protein	Translation	Intracellular	Inflorescence, Root
	P	1.682.111				
At3g57600	NP	-2.521.353	Encodes a member of the DREB subfamily A-2 of	Transcription regulation	Nucleus	Rosette
	P	1.680.785				

			ERF/AP2 transcription factor family			
At1g77815	NP	-1.657.187	Polynucleotidyl transferase, ribonuclease H-like superfamily protein	Unknown	Unknown	Unknown
	P	1.665.614				
At1g28250	NP	-1.675.972	Unknown protein	Unknown	Unknown	Inflorescence, Root
	P	1.661.507				
At5g16260	NP	-2.010.447	RNA binding protein Early Flowering 9 (ELF9)	Flower development, mRNA nonsense-mediated decay	Nucleus	Inflorescence
	P	1.645.528				
At5g16280	NP	-1.924.226	Tetratricopeptide repeat (TPR)-like superfamily protein	Unknown	Endomembrane system	Inflorescence
	P	1.626.150				
At1g68800	NP	-1.735.674	Encodes a TCP transcription factor, closely related to teosinte branched1 (BRC2)	Transcription regulation, secondary shoot formation	Nucleus	Inflorescence
	P	1.622.053				
At5g41130	NP	-2.335.224	Esterase/lipase/thioestera se family protein	Metabolic process	Unknown	Unknown
	P	1.597.087				
At5g40070	NP	-1.869.780	MADS-box family protein	Unknown	Chloroplast	Inflorescence
	P	1.587.226				
At3g58330	NP	-2.090.172	Phospholipase-like protein (PEARLI 4) family	Unknown	Unknown	Inflorescence
	P	1.562.033				
At1g12460	NP	-1.733.917	Leucine-rich repeat protein kinase family protein	Protein phosphorylation	Plasma membrane	Inflorescence
	P	1.561.872				
At3g60580	NP	-1.798.217	Zinc finger (C2H2 type) family protein	Transcription regulation	Nucleus	Inflorescence
	P	1.537.458				

At5g54110	NP	-1.551.241	Highly polar protein with more than 60% hydrophilic amino acid residues, associated with the plasma membrane (MAMI)	Response to osmotic stress	Plasma membrane	Inflorescence
	P	1.514.329				
At4g04790	NP	-1.954.280	Tetratricopeptide repeat (TPR)-like superfamily protein	Unknown	Unknown	Inflorescence
	P	1.506.521				

[a] Polyribosomes (P) and non-polyribosomes (NP) were obtained by sucrose gradient (20-60%) fractionation as described in Materials and methods and shown in Figure S4.

[b] The GeneArise software identified differentially distributed mRNAs in *AteIF(iso)4E-1* fractions considering a z-score > 1.5 standard deviations.

[c] Data were taken from The Arabidopsis Resource Center (TAIR).

[d] The plant structure reported with the higher mRNA level was considered according to the AtGenexpress Visualization Tool (Schmid, M., Davison, T.S., Henz, S.R., et al., 2005, A gene expression map of Arabidopsis thaliana development, Nature Genetics 37: 501-506).
